# Acorenone C: A New Spiro-Sesquiterpene from a Mangrove-Associated Fungus, *Pseudofusicoccum* sp. J003

**DOI:** 10.3389/fchem.2021.780304

**Published:** 2021-11-25

**Authors:** Shujie Jia, Xiangdong Su, Wensi Yan, Meifang Wu, Yichuang Wu, Jielang Lu, Xin He, Xin Ding, Yongbo Xue

**Affiliations:** School of Pharmaceutical Sciences (Shenzhen), Shenzhen Campus of Sun Yat-sen University, Shenzhen, China

**Keywords:** *Pseudofusicoccum* sp., *Sonneratia apetala* Buch.-Ham., sesquiterpenoid, anti-inflammation, acetylcholinesterase

## Abstract

Mangrove-derived endophytes are rich in bioactive secondary metabolites with a variety of biological activities. Recently, a fungus *Pseudofusicoccum* sp. J003 was first isolated by our research group from mangrove species *Sonneratia apetala* Buch.-Ham. The subsequent chemical investigation of the methanol extract of the culture broth of this strain has led to the isolation of a new sesquiterpenoid named acorenone C **(1)**, two alkaloids **(2–3)**, four phenolic compounds **(4–**7**)**, and four steroid derivatives **(8–11)**. The new structure of **1** was established by extensive spectroscopic analysis, including 1D, 2D NMR spectroscopy, and HRESIMS. Its absolute configuration was elucidated by experimental ECD and ECD calculation. The *in vitro* AChE inhibitory, anti-inflammatory, and cytotoxic activities of the selected compounds were evaluated. The results showed that compound **1** showed mild AChE inhibitory activity, with an inhibition rate of 23.34% at the concentration of 50 *μ*M. Compound **9** exerted a significant inhibitory effect against nitric oxide (NO) production in LPS-stimulated RAW 264.7 mouse macrophages, with an inhibition rate of 72.89% at the concentration of 25 *μ*M, better than that of positive control L-NMMA. Compound **9** also displayed obvious inhibition effects on the growth of two human tumor cell lines, HL-60 and SW480 (inhibition rates 98.68 ± 0.97% and 60.40 ± 4.51%, respectively). The antimicrobial activities of the compounds **(1–11)** against *Escherichia coli*, *Bacillus subtilis*, *Staphylococcus aureus*, and *Pseudomonas aeruginosa* were also tested; however, none of them showed antimicrobial activities.

## Introduction

The great diversity of creatures in the ocean was found to be a rich reservoir of candidates for drug development (Sigwart et al., 2021). To date, more than 35,000 marine natural products have already been discovered, which have a higher rate of successful drug discovery than other naturally occurring compounds (Lyu et al., 2021; Sigwart et al., 2021). Mangroves are an intertidal wetland ecosystem spreading across low-latitude tropical and subtropical regions, which are found to have potential to control coastal erosion and protect coastal land. The ingredients produced by mangrove plant species may play a role in helping them survive from universally unfavorable factors (Bandaranayake, 2002). Many types of natural products have been identified from mangroves and their endophytes, including heterocyclic compounds, benzofurans, alkaloids, lignin, polysaccharides, fatty acids, lipids, anthocyanins, flavonoids, phenols and quinones, tannins, limonin, terpenoids, steroids, and saponins (Carroll et al., 2020).

Recently, our research group aimed at structurally diverse natural products from the mangroves and their endophytes for pharmaceutical drug discovery. As a small- to medium-sized columnar true mangrove, the plant species *Sonneratia apetala* Buch.-Ham. is native to South Asia and Southeast Asia and has been cultivated in Guangdong and Hainan provinces, China (Hossain et al., 2016). *S. apetala* has versatile pharmacological effects, for example, the extracts of barks and leaves of *S. apetala* exhibited antibacterial, antioxidant, anti-diabetic, and anti-cancer activities (Patra et al., 2015). However, the endophytes of *S. apetala* were scarcely investigated.

**FIGURE 1:**
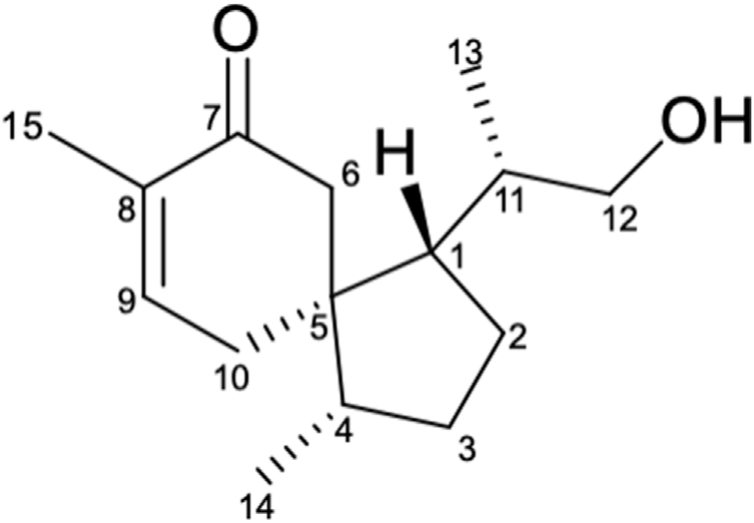
Structure of compound 1.

In this work, a fungus *Pseudofusicoccum* sp. J003 was isolated from the fruit of *S. apetala* for the first time. Previous studies on the secondary metabolites obtained from the genus *Pseudofusicoccum* by other research groups revealed the presence of phenolic compounds (Abba et al., 2018), cyclopeptides, and rotenoids (Sobreira et al., 2018). In our study, the chemical investigation into the methanol extract of this strain by repeated column chromatography over silica gel, Sephadex LH-20, RP-C_18_ silica, and semi-preparative HPLC resulted in the isolation of a new sesquiterpenoid **(1)** (Figure 1), two alkaloids **(2–3)**, four phenolic compounds **(4–7)**, and four steroid derivatives **(8–11)**. Herein, the isolation, structure determination of isolated compounds, and evaluation of their *in vitro* anti-inflammatory, antimicrobial, cytotoxic, and AChE inhibitory activities were described.

## Materials and Methods

### General Experimental Procedures

The optical rotations, CD, and FT-IR spectra were measured with a Perkin-Elmer 341 polarimeter (PerkinElmer, Waltham, MA, USA), JASCO J-810 spectrometer (Jasco Corporation, Japan), Bruker Vertex 70 FT-IR spectrophotometer (Bruker, Karlsruhe, Germany), respectively. The UV spectrum was recorded using a Waters e2695 spectrophotometer (Waters, Massachusetts, USA) equipped with a DAD and a 1-cm-path length cell. Samples in methanol solution were scanned from 190 to 400 nm in 1-nm steps. The structure characterization of the obtained compound was based on 1D NMR (^1^H, ^13^C) and 2D NMR (COSY, HSQC, HMBC, and NOESY) data, recorded on the Bruker AM-400, AM-500, and AM-700 NMR spectrometers (Bruker, Karlsruhe, Germany) with TMS as internal standard, respectively. The detailed parameters for the NMR data of all isolates are provided (see Supporting Information, Supplementary Figures S3–S9; Supplementary Figures S11–S30). Chemical shifts (*δ*) were expressed in ppm with reference to the solvent signals. HRESIMS data were acquired on a Thermo Fisher LTQ XL LC/MS (Thermo Fisher, Palo Alto, CA, USA). Semi-preparative HPLC was performed on an Agilent 1220 apparatus equipped with a UV detector with a semi-preparative column (RP-C_18_, 5 μm, 250 × 10 mm, Welch Materials, Inc.). Column chromatography was performed using silica gel (200–300 mesh and 80–120 mesh, Qingdao Marine Chemical Co., Ltd., Qingdao, China) and Sephadex^TM^ LH-20 gel (40–70 μm; Merck, Darmstadt, Germany). Fractions were monitored by TLC (GF254, Qingdao Marine Chemical Co., Ltd., Qingdao), and spots were visualized by heating silica gel plates sprayed with 10% H_2_SO_4_ in EtOH. All solvents were of analytical grade (Guangzhou Chemical Regents Company, Ltd., Guangzhou, China).

### Fungal Isolation and Fermentation

The fungal strain *Pseudofusicoccum* sp. J003 was isolated from the fruit of *Sonneratia apetala* Buch.-Ham., which was collected at a wetland of Nansha district, Guangzhou, China, in September 2020. The sequence data for this strain have been submitted to the GenBank under accession no. MZ854244. The fungal strain was deposited on 20% aqueous glycerol stock in a −80°C freezer at the School of Pharmaceutical Sciences (Shenzhen), Shenzhen Campus of Sun Yat-sen University, Shenzhen, China. The strain was cultured on potato dextrose agar for 5 days at 28°C. Agar plates, including the strain, were cut into small pieces, and then these pieces were inoculated in a tissue culture bottle (150 × 350 ml) on a solid rice medium (40 g of rice and 35 ml of distilled water) and cultured at room temperature for 30 days.

### Extraction and Isolation of Secondary Metabolites

Cultural media were extracted with methanol three times. Methanol was removed by reduced pressure evaporation at 45°C, and the remaining aqueous phase was extracted 4 times with ethyl acetate. The ethyl acetate layer was concentrated under reduced pressure to yield a brown extract (60.0 g). The crude extract was introduced to a silica gel chromatography column (CC) and eluted with petroleum ether/ethyl acetate (35:1→0:1) to obtain seven fractions (Fr. 1–Fr. 7). Fr. 2 (7.3 g) was separated into 7 subfractions (Fr. 2.1–Fr. 2.7) using silica gel CC and eluted with *n*-hexane/2-propanol. Fr. 2.2 (102.3 mg) was purified by semi-preparative HPLC (100% MeOH, 3.0 ml/min) to yield 4 (21.6 mg, *t*_R_ 24.5 min). Fr. 2.6 (800.5 mg) was purified by semi-preparative HPLC (45% MeOH/H_2_O, v/v, 3.0 ml/min) to yield 5 (1.3 mg, *t*_R_ 8.5 min). Fr. 2.4 (113.0 mg) and Fr. 2.7 (220.7 mg) were separated by repeated CC over silica gel to yield 10 (3.4 mg) and 9 (6.7 mg). Fr. 4 (500.5 mg) was separated with repeated silica gel CC to yield six fractions (Fr. 4.1–Fr. 4.6) and then subjected subfraction Fr. 4.3 (268.3 mg) to a Sephadex LH-20 CC (CHCl_2_–MeOH, 1:1) to afford three parts (Fr. 4.4a–Fr. 4.4c). Fr. 4.4b (43.2 mg) was purified by semi-preparative HPLC (100% MeOH/H_2_O, v/v, 3.0 ml/min) to yield 8 (14.0 mg, *t*_R_ 14.5 min). Fr. 4.4c (55.8 mg) was purified by semi-preparative HPLC (70% MeOH/H_2_O, v/v, 3.0 ml/min) to yield 1 (2.0 mg, *t*_R_ 23.1 min). Fr. 5 (18.9 g) was separated with repeated Sephadex LH-20 CC (MeOH) to yield 6 (7.1 mg) and 7 (90.0 mg). Fr. 7 (7.7 g) was separated with repeated silica gel CC and eluted with CH_2_Cl_2_/MeOH and *n*-hexane/2-propanol to yield 2 (22.0 mg), 3 (20.0 mg), and 11 (10.0 mg).

Acorenone C 1) Colorless oil; ${\left[\alpha \right]}_{D}^{29}$ –34.6 (*c* 0.1, MeOH); UV (MeOH) *λ*_max_ (log *ε*) 233 (1.2) nm; CD (0.10 mM, MeOH) *λ*_max_ (Δε) 213 (−1.72), 244 (+9.25) nm; IR *v*_max_ 3,429, 2,951, 2,922, 2,872, 1,663, 1,456, 1,381, 1,369, 1,248, 1,034 cm^−1^; ^1^H NMR and ^13^C NMR data (see Table 1 **)**; HRESIMS [M + Na]^+^ *m/z* 259.1679 (calcd. for C_15_H_24_O_2_Na, 259.1669).

**TABLE 1:** ^1^H and ^13^C NMR data for 1 (Record in CD_3_OD, *J* in Hz).

No.	*δ*_*H*_	*δ*_*C*_
1	1.66 m	52.2
2a	1.54 m	23.6
2b	1.65 m	
3a	1.32 m	30.8
3b	1.83 m	
4	1.67 m	46.6
5		50.0
6a	2.63 d (16.5)	49.8
6b	2.24 d (16.5)	
7		203.2
8		136.1
9	6.82 t like (3.9)	147.1
10a	2.36 dm (19.4)	27.4
10b	2.22 dm (19.4)	
11	1.76 m	36.3
12a	3.36 m	68.5
12b	3.38 m	
13	0.91 d (6.7)	14.7
14	0.84 d (6.8)	17.4
15	1.75 s	15.4

### Anti-AChE Assay

Acetylcholinesterase (AChE) inhibitory activity of the compounds isolated was assayed by the spectrophotometric method with slight modification (Ellman et al., 1961). *S*-Acetylthiocholine iodide, *S*-butyrylthiocholine iodide, 5,5′-dithio-bis-(2-nitrobenzoic) acid (DTNB, Ellman’s reagent), and acetylcholinesterase derived from human erythrocytes were purchased from Sigma Chemical. The compounds were dissolved in DMSO. The reaction mixture (totally 200 μL) containing phosphate buffer (pH 8.0), a test compound (50 μM), and acetyl cholinesterase (0.02 U/mL) was incubated for 20 min (37°C). Then the reaction was initiated by the addition of 40 μL of a solution containing DTNB (0.625 mM) and acetylthiocholine iodide (0.625 mM) for AChE inhibitory activity assay, respectively. The hydrolysis of acetylthiocholine was monitored at 405 nm every 30 s for 1 h. Tacrine was used as a positive control with a final concentration of 0.333 μM. All the reactions were performed in triplicate. The percentage inhibition was calculated as follows: % inhibition = (E–S)/E × 100 (E is the activity of the enzyme without the test compound and S is the activity of the enzyme with the test compound).

### Anti-Inflammatory Assay

The RAW 264.7 cells (2 × 10^5^ cells/well) were incubated in 96-well culture plates with or without 1 μg/ml LPS (Sigma Chemical Co., USA) for 24 h in the presence or absence of the test compounds. Aliquots of supernatants (50 µL) were then reacted with 100 µL Griess reagent (Sigma Chemical Co., USA). The absorbance was measured at 570 nm by using the Synergy TMHT Microplate Reader (BioTek Instruments Inc., USA). In the study, L-NMMA (Sigma Chemical Co., USA) was used as a positive control. In the remaining medium, an MTT assay was carried out to determine whether the suppressive effect was related to cell viability. The inhibitory rate of NO production = (NO level of blank control – NO level of test samples)/NO level of blank control. The percentage of NO production was evaluated by measuring the amount of nitrate concentration in the supernatants with Griess reagent, as described previously (Wu et al., 2017).

### Cytotoxicity Assay

Five human cancer cell lines, including the A549 lung cancer cell line, the HL-60 human myeloid leukemia cell line, the MCF-7 breast cancer cell line, the SMMC-7721 human hepatocarcinoma cell line, and the SW-480 human pancreatic carcinoma were used. Cells were cultured in RPMI-1640 or DMEM medium, supplemented with 10% fetal bovine serum and 5% CO_2_ at 37°C. The cytotoxicity assay was performed using an MTTS 3-(4,5-dimethylthiazol-2-yl)-5(3-carboxymethoxyphenyl)-2-(4-sulfopheny)-2H-tetrazolium) method in 96-well microplates, as reported previously (Liu et al., 2012), with slight modification. In brief, 100 μL of adherent cells were seeded into each well of the 96-well culture plates and allowed to adhere for 12 h before adding the test compounds, while suspended cells were seeded into wells at a density of 1 × 10^5^ cells/mL just prior to the addition of the test compounds. Each tumor cell line was exposed to the test compound at concentrations of 40 μM in triplicates for 48 h. Wells with DMSO were used as negative controls, and Taxol and DDP were used as positive controls. After treatment of the compounds, cell viability was detected by a microplate reader at *λ* = 492 nm.

### Antimicrobial Assay

Compounds 1–11 were evaluated for their antimicrobial activities against *Escherichia coli*, *Bacillus subtilis*, *Staphylococcus aureus*, and *Pseudomonas aeruginosa*. The antimicrobial assay was conducted by the previously described method (Zhang et al., 2019). The sample to be tested was added into a 96-well culture plate, and the maximum concentration of the used compounds was 250 μg/ml. Bacteria liquid was added to each well until the final concentration is 5 × 10^5^ CFU/ml. It was then incubated at 37°C for 24 h, and the OD value at 595 nm was measured by the microplate reader, and the medium blank control was used in the experiment.

## Results and DISCUSSION

### Identification of Compounds

Compound 1 was obtained as colorless oil. Its molecular formula was determined to be C_15_H_24_O_2_ based on the deprotonated ion peak [M + Na]^+^ at *m/z* 259.1679 [M + Na]^+^ (calcd for 259.1669) in the (+)-HRESIMS, indicating 4 degrees of unsaturation. The IR spectrum showed characteristic absorption bands of hydroxyl (3,429 cm^−1^) and the carbonyl groups (1,662 cm^−1^). The ^13^C NMR and DEPT spectra of **1** (Table 1) showed 15 carbon signals, including three methyls, five methylenes (including oxygenated methylene at *δ*c 68.5), three methine groups, two olefinic carbon signals (*δ*c 147.1 and 136.1), an aliphatic quaternary carbon, and a carbonyl carbon (*δ*_C_ 203.2). The ^1^H NMR spectrum of 1 (Table 1) displayed the presence of an olefinic proton resonated at *δ*_H_ 6.82 (t like, *J* = 3.9 Hz), two methyl group doublets at 0.91 (d, *J* = 6.7 Hz) and 0.84 (d, *J* = 6.8 Hz), and two oxygenated methine protons at *δ*_H_ 3.36 and 3.38. Apart from the two degrees of unsaturation occupied by the carbonyl group and a double bond, the remaining degrees of unsaturation suggested that compound **1** should be a dicyclic sesquiterpenoid (Amandine et al., 2017).

**FIGURE 2:**
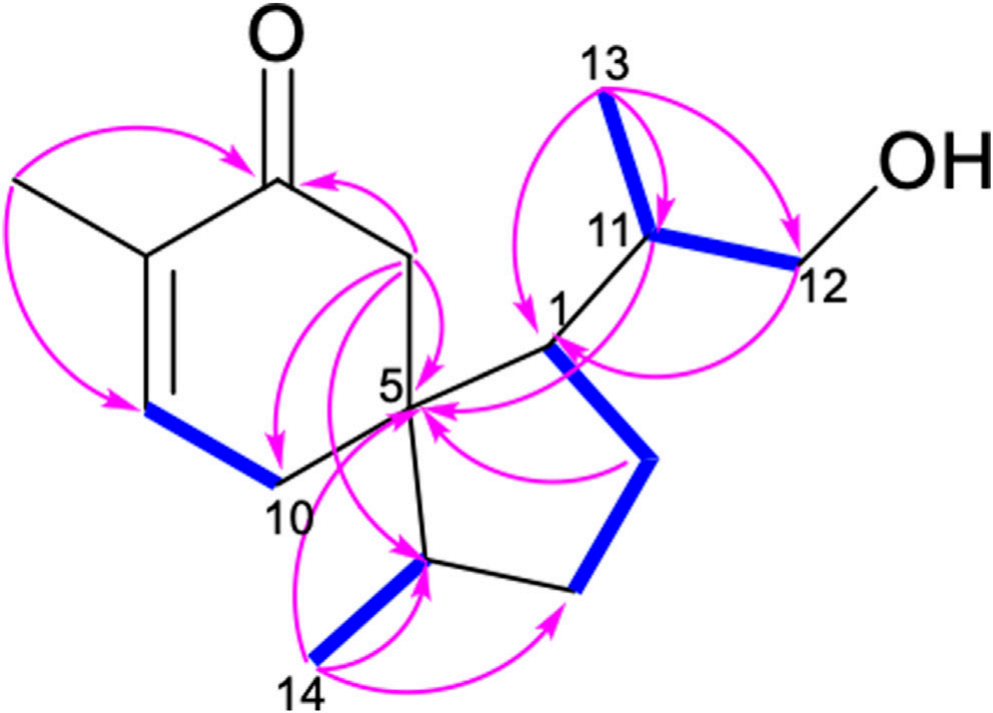
Key COSY (bolds, blue) and HMBC (arrows, pink) correlations of 1.

The ^1^H-^1^H COSY spectrum of **1** indicated the presence of spin systems of (HO)CH_2_(12)-CH(11)-CH_3_(13) and CH(1)-CH_2_(2)-CH_2_(3)-CH(4)-CH_3_(14) (Figure 2). In the HMBC spectrum, the HMBC interactions from H-13 (*δ*_H_ 0.91) and H-12 (*δ*_H_ 3.36 and 3.38) to C-11 (*δ*_C_ 36.3) and C-1 (*δ*_C_ 52.2) revealed the direct C–C linkage from C-11 to C-1 (Figure 2). Subsequently, the HMBC correlations of H-2 and H-11 with C-5 (*δ*_C_ 50.0) and of H-14 with C-3, C-4, and C-5 indicated the presence of a methyl cyclopentane substructure with a 1-propanol substituted at C-1. The spin system of = CH(9)–CH_2_(10) observed from the ^1^H–^1^H COSY spectrum of 1, together with the key HMBC correlations from H_3_-15 (*δ*_H_ 1.75) to C-7 (*δ*_C_ 203.2)/C-8 (*δ*_C_ 136.1)/C-9 (*δ*_C_ 147.1), from H-6 (*δ*_H_ 2.24 and 2.63) to C-5/C-8/C-10, collaborated with the methyl cyclohexane substructure decorated by an *α*,*β*-unsaturated ketone functionality. Based on the aforementioned pieces of evidence, the crucial HMBC correlations from H-6 and H-10 to C-1, C-4, and C-5 and from H-1 and H-4 to C-5, C-6, and C-10 constructed the gross structure of **1**, featuring a spiro[4,5]decane scaffold. The planar structure of 1 was thus deduced as shown (Figure 2), resembling the (3*S*)-1,4-epi-3-hydroxyacorenone (Calva et al., 2017).

**FIGURE 3:**
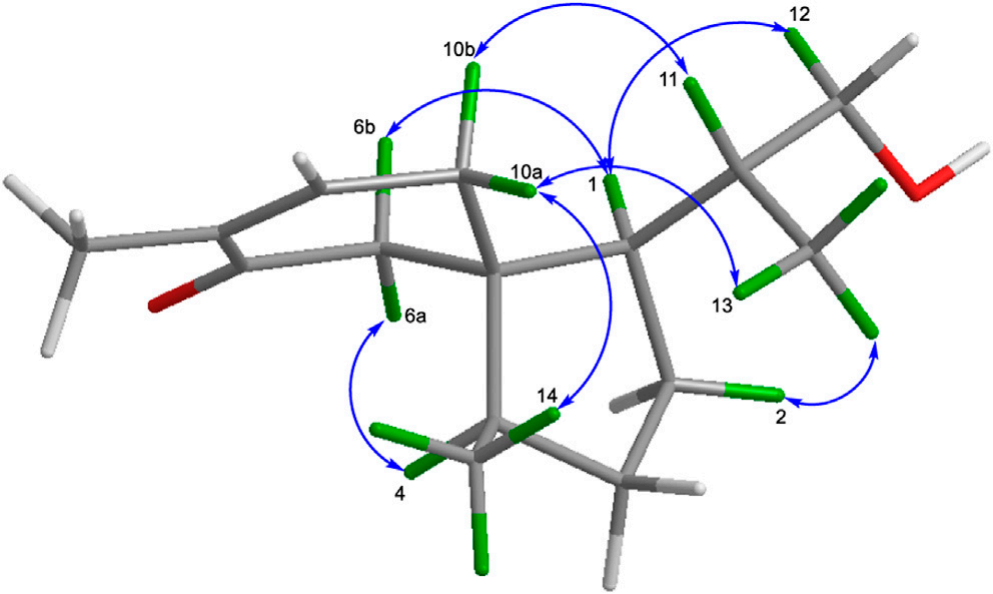
Key NOESY correlations of compounds 1.

**FIGURE 4:**
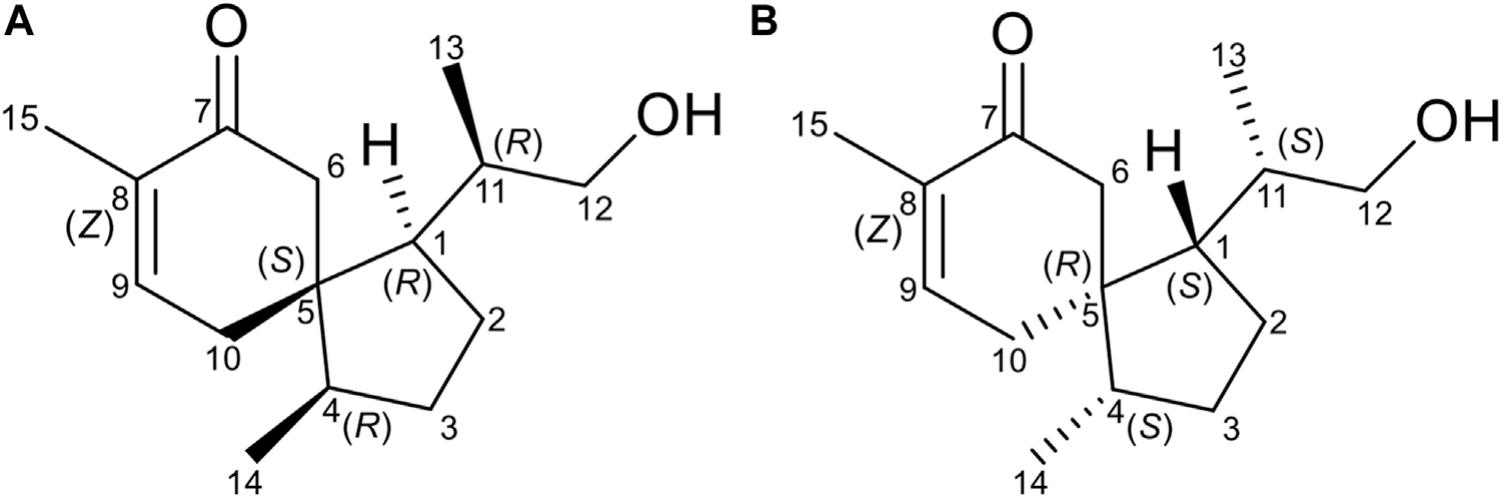
Two possible enantiomers of compound 1 [**(A)** (1*R*,4*R*,5*S*,11*R*) and **(B)** (1*S*,4*S*,5*R*,11*S*)].

The relative configurations of **1** were elucidated by the observation of its NOESY spectrum. The NOESY correlations of H-10a with H_3_-13 and H_3_-14, and Me-13/H-2a revealed that H-10a, H_3_-13, and H_3_-14 were co-facial and provisionally assigned to be *α*-oriented. Accordingly, the NOESY cross-peaks of H-11/H-10b, H-1/H-6b, H-6a/H-4, and H-1/H-12a indicated the *β*-orientation of H-1, H-4, and H-11. The relative stereochemistries at C-1, C-4, C-5, and C-11 of **1** were thus determined (Figure 3). Therefore, as for the absolute configuration of **1**, two possible enantiomers (Figures 4A,B) were presented (Figure 4).

**FIGURE 5:**
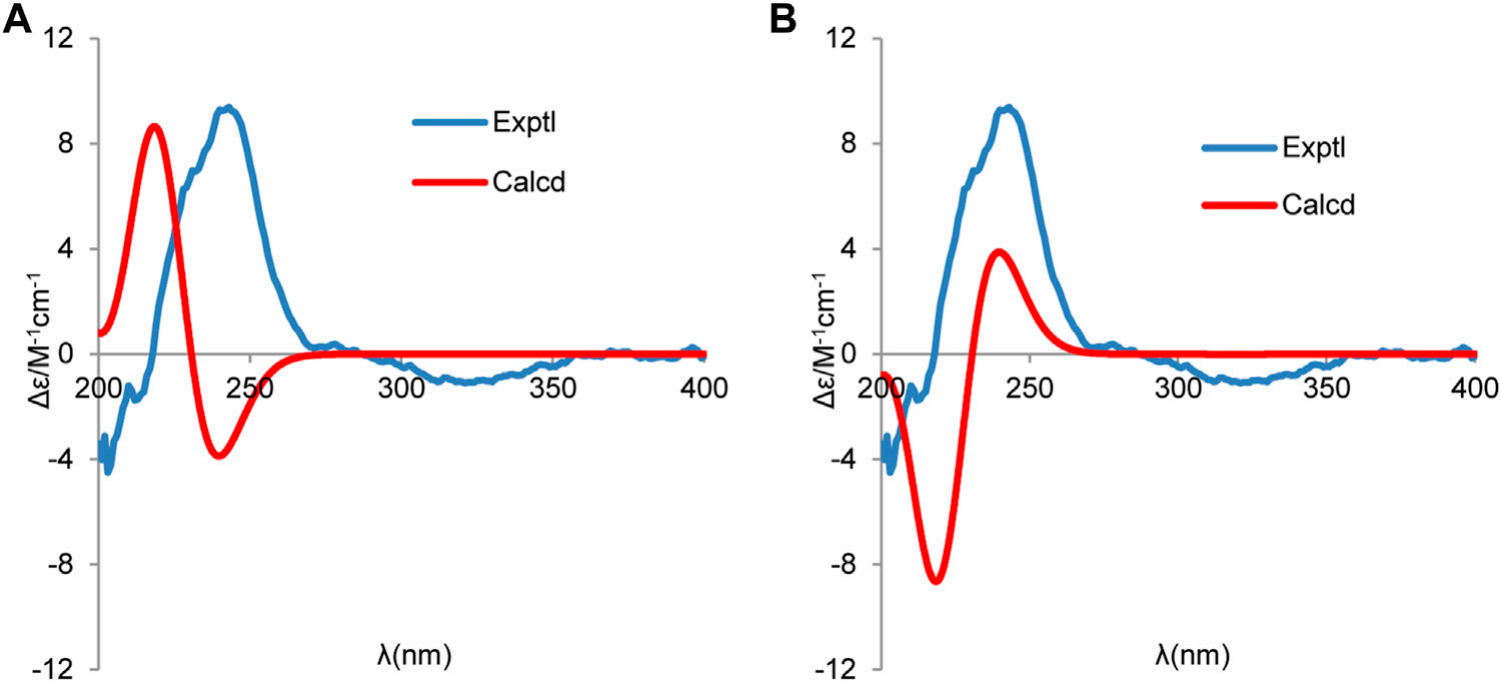
Experimental and calculated ECD spectra of **(A)** (1*R*,4*R*,5*S*,11*R*) and **(B)** (1*S*,4*S*,5*R*,11*S*) (red, calculated at the B3LYP-PCM/6-31G(d,p)//B3LYP/6-31G (d,p) level in CH_3_OH; blue, experimental in CH_3_OH).

To further determine the absolute stereochemistry of **1**, the electronic circular dichroism (ECD) (for detailed procedures, see SI) calculation of the two possible enantiomers (Figures 5A,B; Figures 4, 5) was performed using Gaussian 09 and figured using GaussView 5.0 (Dennington et al., 2009; Frisch et al., 2009). Conformation search *via* molecular mechanics calculations was conducted in Discovery Studio 3.5 Client, with an MMFF force field with a 20 kcal/mol upper energy limit (Smith and Goodman, 2010). The optimized conformation geometries and thermodynamic parameters of the selected conformations were provided. The predominant conformers were subsequently optimized at the B3LYP/6-31G(d,p) level. The theoretical calculation of ECD was performed using a time-dependent density functional theory (TDDFT) at the B3LYP/6-31G(d,p) level in MeOH with the PCM model. The calculated spectrum of 1b (1*S*,4*S*,5*R*,11*S*) agreed with the experimental data, showing a negative Cotton effect (CE) at 213 nm and a strong positive CE at 244 nm (Figure 5). Consequently, the structure of **1** was determined to be (1*S*,4*S*,5*R*)-1-((*S*)-1-hydroxypropan-2-yl)-4,8-dimethylspiro [4.5]dec-8-en-7-one, and a trivial acorenone C was given.

Ten known compounds, uracil **(2)** (Xing et al., 2020), cyclo-(L-Pro-L-Tyr) **(3)** (Jayatilake et al., 1996), bis-(2-ethylhexyl) terephthalate **(4)** (Firdovsi et al., 2007), 4-hydroxybenzaldehyde **(5)** (Shataer et al., 2020), 2-phenylethanol **(6)** (Guerrini et al., 2011), 4-hydroxyphenethyl-alcohol **(7)** (Wei et al., 2013), estigmast-4-en-6β-ol-3-ona **(8)** (Correia et al., 2003), ergosterol **(9)** (Zhang et al., 2002), ergosterol peroxide **(10)** (Hybelbauerová et al., 2008), and cerevisterol **(11)** (Kang et al., 2017) were also isolated from *Pseudofusicoccum* sp. J003. The structures of these compounds **(2–11)** were elucidated by comparing the spectral data to those reported in the references.

### Biological Activity

According to the literature, acorenone analogs usually have AChE inhibitory activity (Calva et al., 2017). The AChE inhibition effect of new compound **1** was tested. It exhibited mild inhibitory activity against AChE with an inhibition rate of 23.34% ± 3.53 at the concentration of 50 μM (Table 2). To further test *in vitro* anti-inflammatory activity, compounds **1–4**, **6–9**, and **11** were evaluated for their inhibitory activities against LPS-induced nitric oxide (NO) production in RAW 264.7 mouse macrophages, of which compound **9** showed obvious inhibitory activity, with an inhibition rate of 72.89% ± 0.71 at the concentration of 25 μM (Table 3). Since steroid derivatives were reported to have cytotoxic properties against tumor cells (Bok et al., 1999), compounds **8**, **9**, and **11** were selected to test their cytotoxic activities against five human cancer cell lines, including HL-60, A549, MCF-7, SMMC-7721, and SW480, of which compound **9** inhibited the proliferation of tumor cells HL-60, with an inhibition rate of 98.68% ± 0.97 and SW480 with an inhibition rate of 60.40% ± 4.51 at a concentration of 40 μM, respectively (Table 4). The antimicrobial activity of compounds **1–11** was also evaluated against the bacteria *S. aureus*, *B. subtilis*, *P. aeruginosa*, and *E. coli*. However, all of them were found to be devoid of significant activity (MIC >250 μg/ml).

**TABLE 2:** AChE inhibitory activity of compound 1.

Compound	Concentration (*μ*M)	Inhibition (%)^a^
**1**	50	23.34 ± 3.53
Tacrine^b^	0.333	58.99 ± 1.67

a All compounds examined in a set of triplicated experiment.

b Positive control.

**TABLE 3:** Inhibitory activities of compounds **1**–**4**, **6**–**9**, and **11** on LPS-stimulated NO production.

Compound	Concentration (μM)	NO production inhibition (%)^a^
**1**	50	−1.05 ± 1.24
**2**	50	−3.51 ± 1.67
**3**	50	−0.18 ± 2.74
**4**	50	−9.74 ± 2.67
**6**	50	6.14 ± 0.66
**7**	50	−3.33 ± 2.19
**8**	50	−1.58 ± 0.79
**9**	25	72.89 ± 0.71
**11**	50	3.16 ± 1.58
L-NMMA^b^	50	52.59 ± 0.99

a All compounds examined in a set of triplicated experiment.

b Positive control.

**TABLE 4:** *In vitro* cytotoxic activity (cell inhibition (%)) of compounds **8**, **9**, and **11** against five human tumor cell lines^a^

Compound	Concentration (*μ*M)	HL-60	A-549	SMMC-7721	MCF-7	SW480
**8**	40	27.90 ± 3.58	49.58 ± 0.49	35.73 ± 1.37	9.26 ± 1.67	15.06 ± 1.99
**9**	40	98.68 ± 0.97	48.25 ± 1.14	46.26 ± 1.63	21.92 ± 1.61	60.40 ± 4.51
**11**	40	20.22 ± 3.11	7.00 ± 2.01	27.91 ± 1.05	21.17 ± 3.50	10.87 ± 0.36
DDP^b^	40	79.06 ± 0.38	84.65 ± 1.00	82.78 ± 0.73	63.55 ± 2.90	78.73 ± 0.62
Taxol^b^	5	54.62 ± 0.46	53.00 ± 0.50	74.50 ± 0.43	58.63 ± 0.58	61.72 ± 2.15

a All compounds examined in a set of triplicated experiment.

b Positive control.

## Conclusion

Mangroves with significant ecological significance and biodiversity have attracted broad interest from scientific communities. In this research, a new sesquiterpenoid called acorenone C **(1)**, along with ten known compounds **(2–11)**, was identified from the culture medium of an endophyte *Pseudofusicoccum* sp. J003, a fungus isolated from a mangrove species *S. apetala*. In addition, compounds **1–6** and **8–11** were identified from the genus *Pseudofusicoccum* for the first time. Their structures were established by extensive spectroscopic analyses, including 1D, 2D NMR spectroscopy, and HRESIMS, as well as ECD calculation. In the vitro bioassays, compound **1** showed mild AChE inhibitory activity, with an inhibition rate of 23.34% at the concentration of 50 *μ*M. Compound **9** exerted a significant inhibitory effect against nitric oxide (NO) production in LPS-stimulated RAW 264.7 mouse macrophages, with an inhibition rate of 72.89% at the concentration of 25 *μ*M, better than that of positive control L-NMMA. Compound **9** also displayed obvious inhibition effects on the growth of two human tumor cell lines HL-60 and SW480 (inhibition rates of 98.68 ± 0.97% and 60.40 ± 4.51%, respectively). The antimicrobial activities of the compounds **(1–11)** against *Escherichia coli*, *Bacillus subtilis*, *Staphylococcus aureus*, and *Pseudomonas aeruginosa* were also tested; however, none of them showed antimicrobial activities. This work will add new bioactive marine natural products from microbes of mangrove plants.

## Data Availability

The datasets presented in this study can be found in online repositories. The names of the repository/repositories and accession number(s) can be found below: https://www.ncbi.nlm.nih.gov/genbank/ MZ854244
