# Supplementary material for: Acorenone C: A New Spiro-Sesquiterpene from a Mangrove-Associated Fungus, Pseudofusicoccum sp. J003
Source: Front Chem. 2021 Nov 25;9:780304. doi: 10.3389/fchem.2021.780304 (PMC8655724; doi:10.3389/fchem.2021.780304)
Supplement: Supplementary file 1 [file DataSheet1.pdf]

Supplementary Material

Acorenone C: A new spiro-sesquiterpene from a Mangrove-associated Fungus, *Pseudofusicoccum* sp. J003

Shujie Jia[1], Xiangdong Su[1], Wensi Yan[1], Meifang Wu[1], Yichuang Wu[1], Jielang Lu[1], Xin He[1], Xin Ding[1], Yongbo Xue[1,*]

[1]School of Pharmaceutical Sciences (Shenzhen), Shenzhen Campus of Sun Yat-sen University, Shenzhen 518107, P. R. China

*** Correspondence:**

Yongbo Xue

xueyb@mail.sysu.edu.cn

List of Supporting Information

Content Page

Figure S1. HRESIMS spectrum of compound **1** 4
Figure S2. IR spectrum of compound **1** 5
Figure S3. The ^{1}H-NMR spectrum of compound **1** (in CD_3OD, 400 MHz) 6
Figure S4. The ^{13}C-NMR and partially enlarged spectrum of compound **1** (in CD_3OD, 100 MHz) 7
Figure S5. The HSQC spectrum of compound **1** (in CD_3OD, 400 MHz) 8
Figure S6. The COSY spectrum of compound **1** (in CD_3OD, 400 MHz) 9
Figure S7. The HMBC spectrum of compound **1** (in CD_3OD, 400 MHz) 10
Figure S8. The ^{1}H-NMR spectrum of compound **1** (in CD_3OD, 500 MHz) 11
Figure S9. The NOESY spectrum of compound **1** (in CD_3OD, 500 MHz) 12
Figure S10. Structures of the co-isolated known compounds **2-11** 13
Figure S11. The ^{1}H-NMR spectrum of compound **2** (in DMSO, 600 MHz) 14
Figure S12. The ^{13}C-NMR spectrum of compound **2** (in DMSO, 150 MHz) 15
Figure S13. The ^{1}H-NMR spectrum of compound **3** (in $CDCl_3$, 400 MHz) 16
Figure S14. The ^{13}C-NMR spectrum of compound **3** (in $CDCl_3$, 100 MHz) 17
Figure S15. The ^{1}H-NMR spectrum of compound **4** (in CD3OD, 400 MHz) 18
Figure S16. The ^{13}C-NMR spectrum of compound **4** (in CD3OD, 100 MHz) 19
Figure S17. The ^{1}H-NMR spectrum of compound **5** (in CD3OD, 700 MHz) 20
Figure S18. The ^{13}C-NMR spectrum of compound **5** (in CD3OD, 176 MHz) 21
Figure S19. The ^{1}H-NMR spectrum of compound **6** (in $CDCl_3$, 600 MHz) 22
Figure S20. The ^{13}C-NMR spectrum of compound **6** (in $CDCl_3$, 150 MHz) 23
Figure S21. The ^{1}H-NMR spectrum of compound **7** (in CD3OD, 400 MHz) 24
Figure S22. The ^{13}C-NMR spectrum of compound **7** (in CD3OD, 100 MHz) 25
Figure S23. The ^{1}H-NMR spectrum of compound **8** (in $CDCl_3$, 400 MHz) 26
Figure S24. The ^{13}C-NMR spectrum of compound **8** (in $CDCl_3$, 100 MHz) 27
Figure S25. The ^{1}H-NMR spectrum of compound **9** (in $CDCl_3$, 400 MHz) 28
Figure S26. The ^{13}C-NMR spectrum of compound **9** (in $CDCl_3$, 100 MHz) 29
Figure S27. The ^{1}H-NMR spectrum of compound **10** (in $CDCl_3$, 400 MHz) 30
Figure S28. The ^{13}C-NMR spectrum of compound **10** (in $CDCl_3$, 100 MHz) 31
Figure S29. The ^{1}H-NMR spectrum of compound **11** (in DMSO, 700 MHz) 32
Figure S30. The ^{13}C-NMR spectrum of compound **11** (in DMSO, 176 MHz) 33

Figure S31. Optimized geometries of predominant conformers for compound **1a** at the B3LYP/6-31G (d,p) level in the gas phase 34

Figure S32. Calculated and experimental ECDs of **1a** and **1b** (red, calculated at the B3LYP-PCM/6-31G (d,p)//B3LYP/6-31G (d,p) level in CH_3OH; blue, experimental in CH_3OH)...............................36

Table S1. Important thermodynamic parameters (a. u.) and Boltzmann distributions of the optimized compound **1a** at B3LYP/6-31G (d,p) level in the gas phase 37

Table S2. Optimized Z-matrixes of compound **1a** in the gas phase (Å) at B3LYP/6-31G (d,p) level 38

Figure S1. HRESIMS spectrum of compound **1**

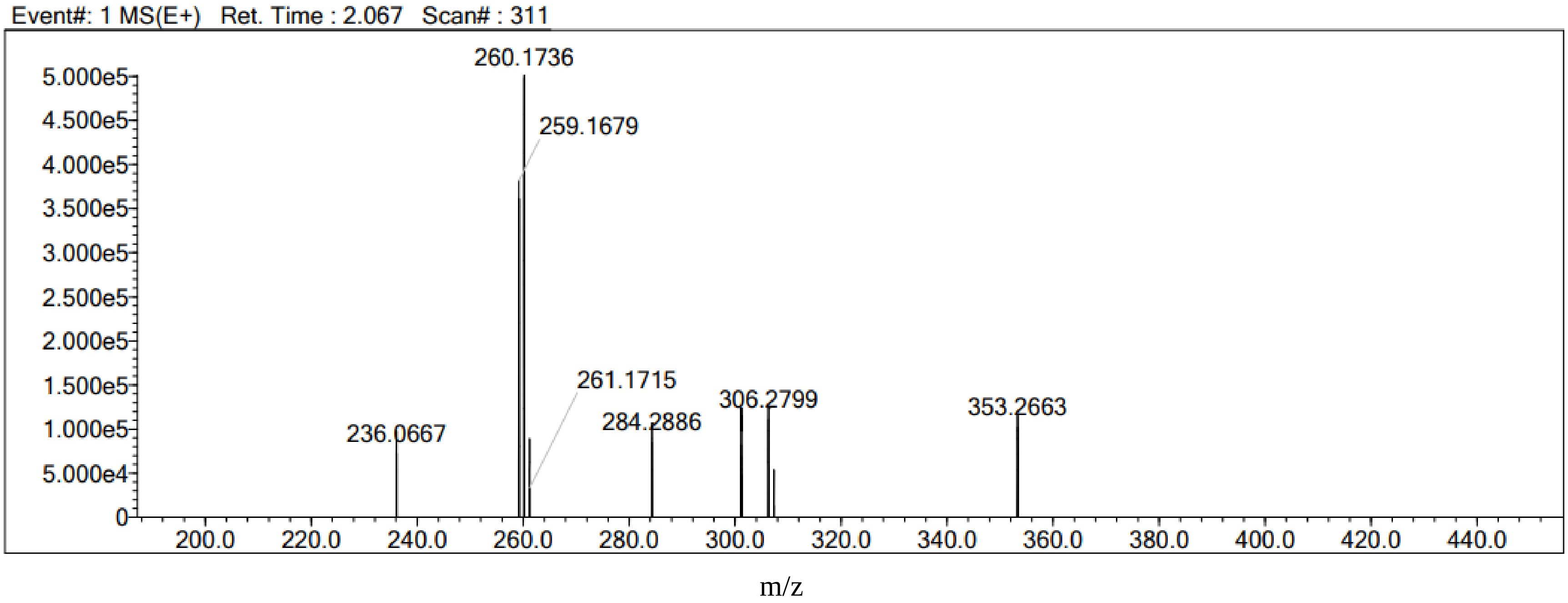

Figure S2. IR spectrum of compound **1**

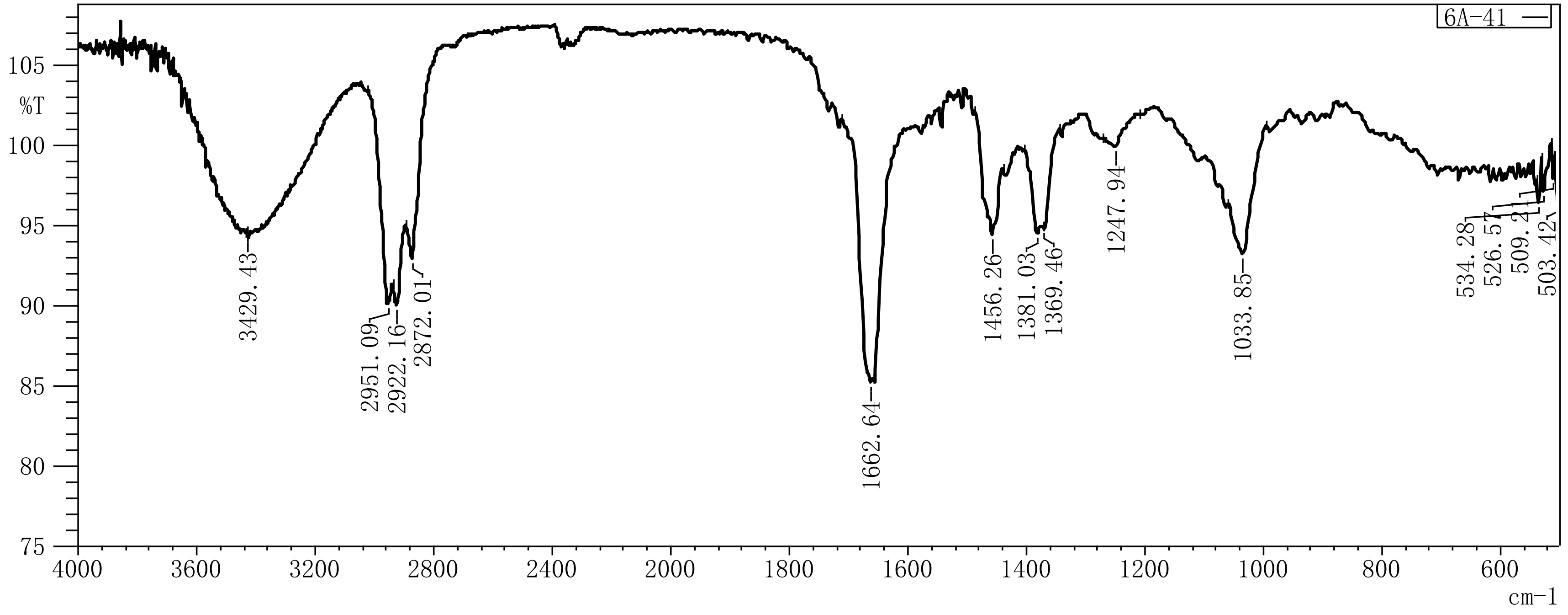

Figure S3. The ^{1}H-NMR spectrum of compound **1** (in CD_3OD, 400 MHz)

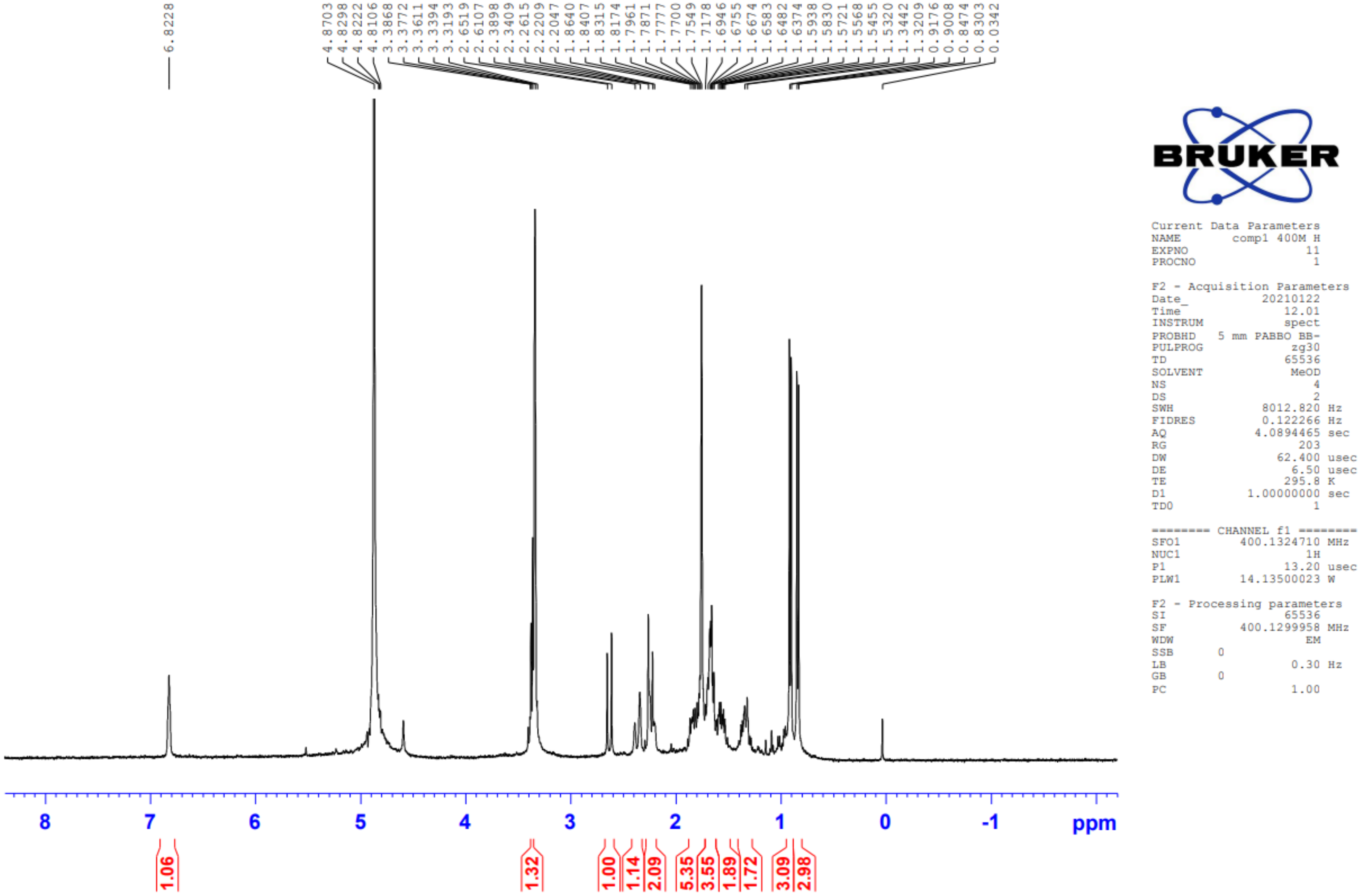

Figure S4. The ^{13}C-NMR and partially enlargedspectrum of compound 1 (in CD_3OD, 100 MHz)

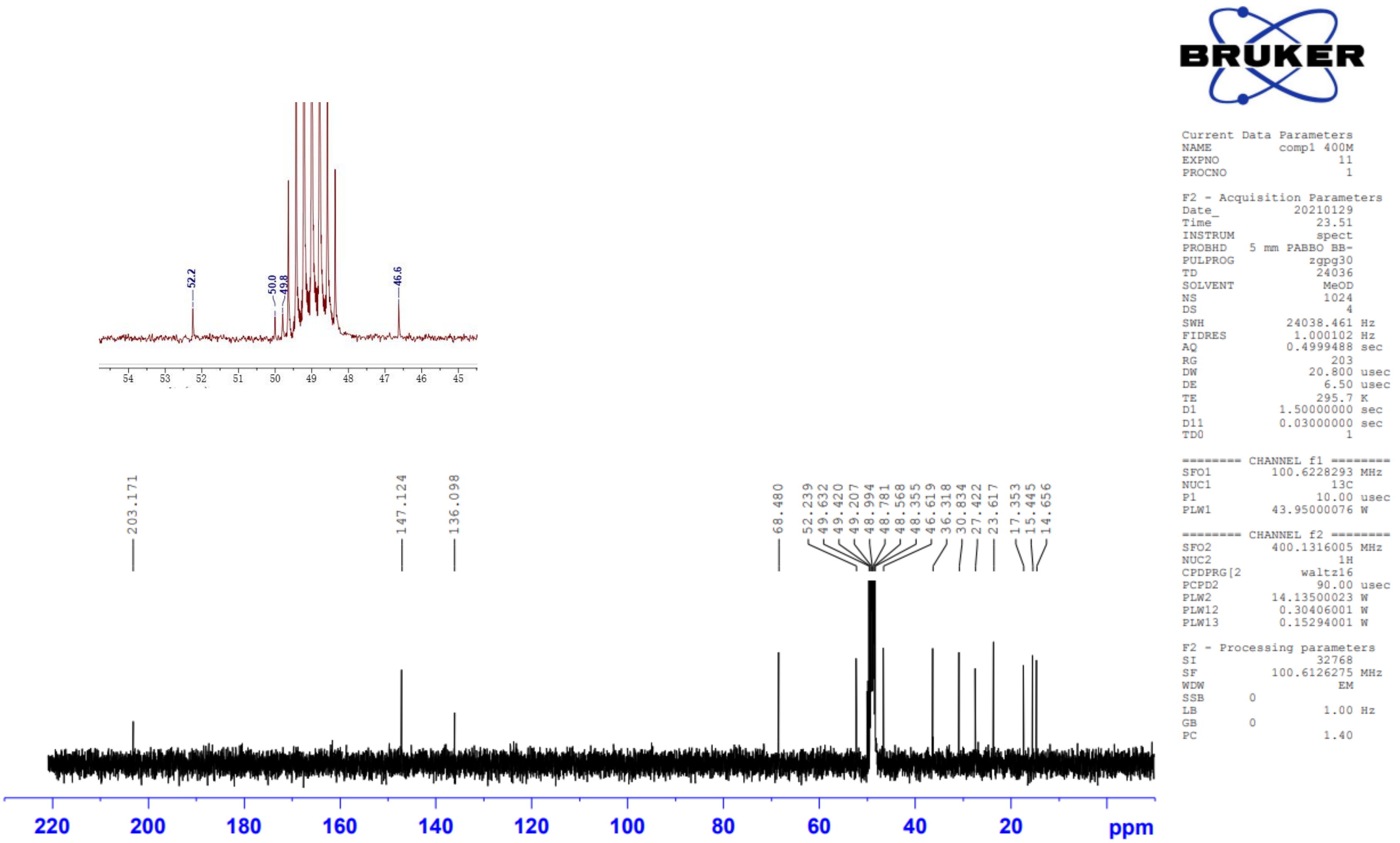

Figure S5. The HSQC spectrum of compound 1 (in CD_3OD, 400 MHz)

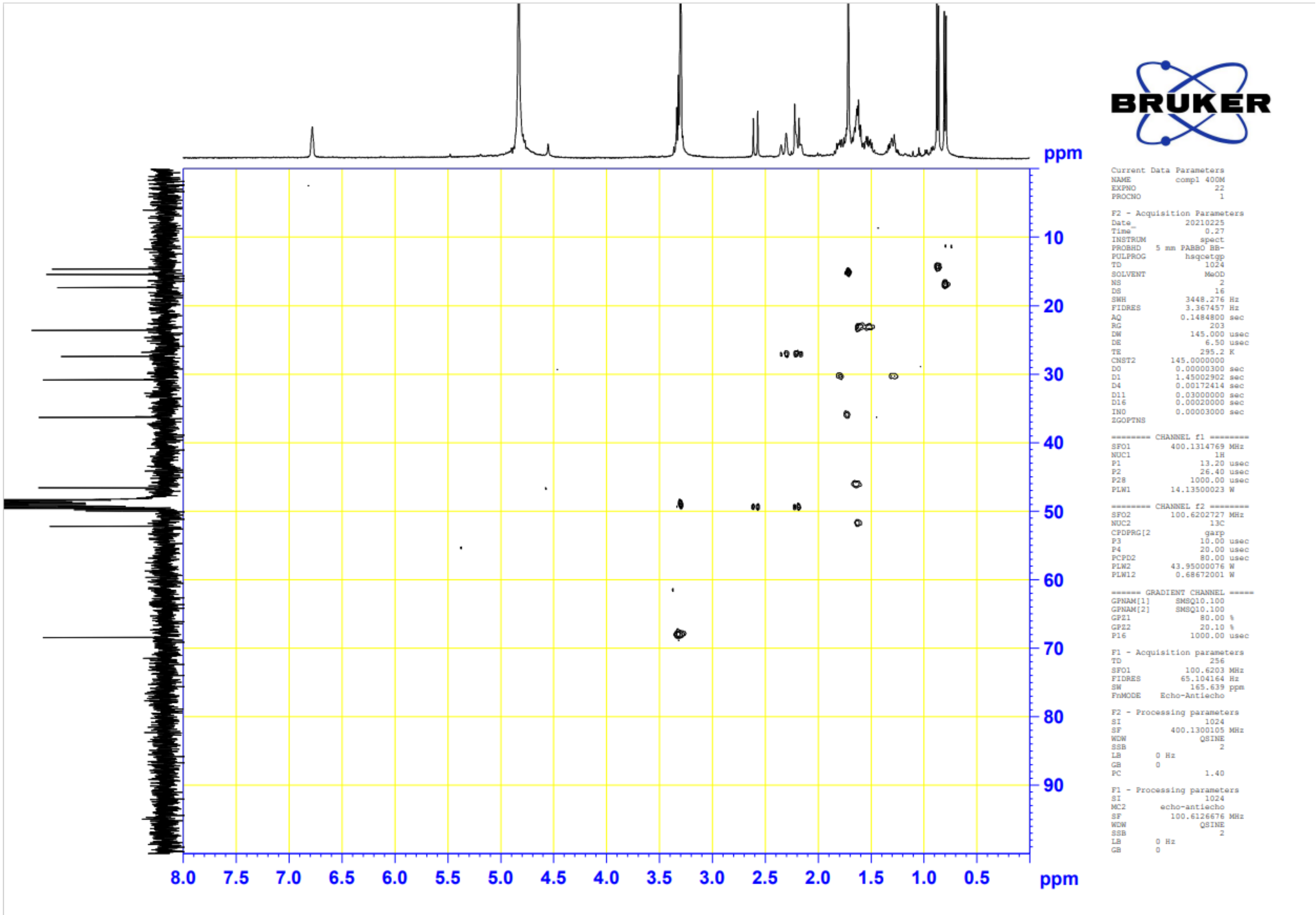

Figure S6. The COSY spectrum of compound 1 (in CD_3OD, 400 MHz)

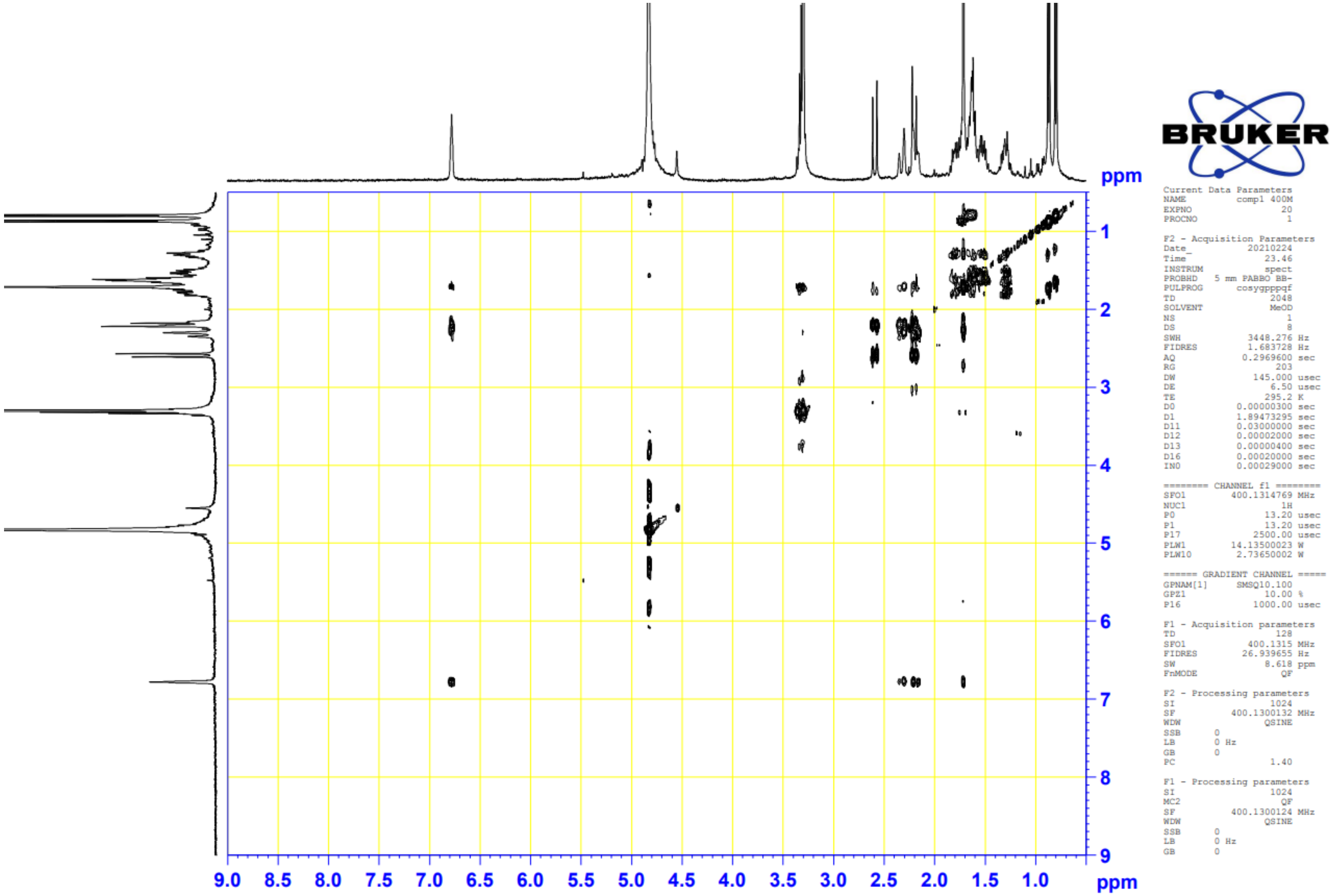

Figure S7. The HMBC spectrum of compound 1 (in CD_3OD, 400 MHz)

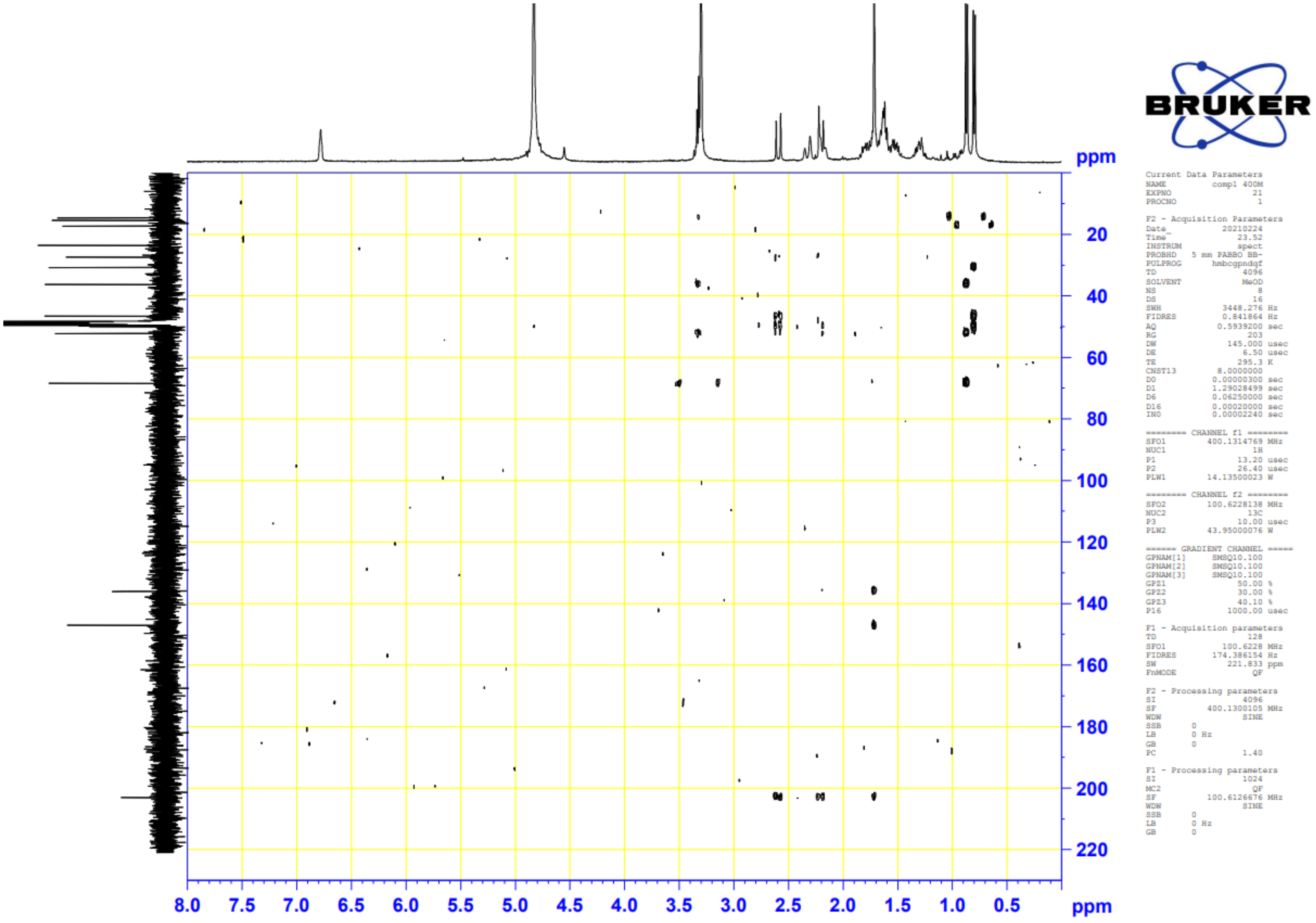

Figure S8. The ^{1}H-NMR spectrum of compound 1 (in CD_3OD, 500 MHz)

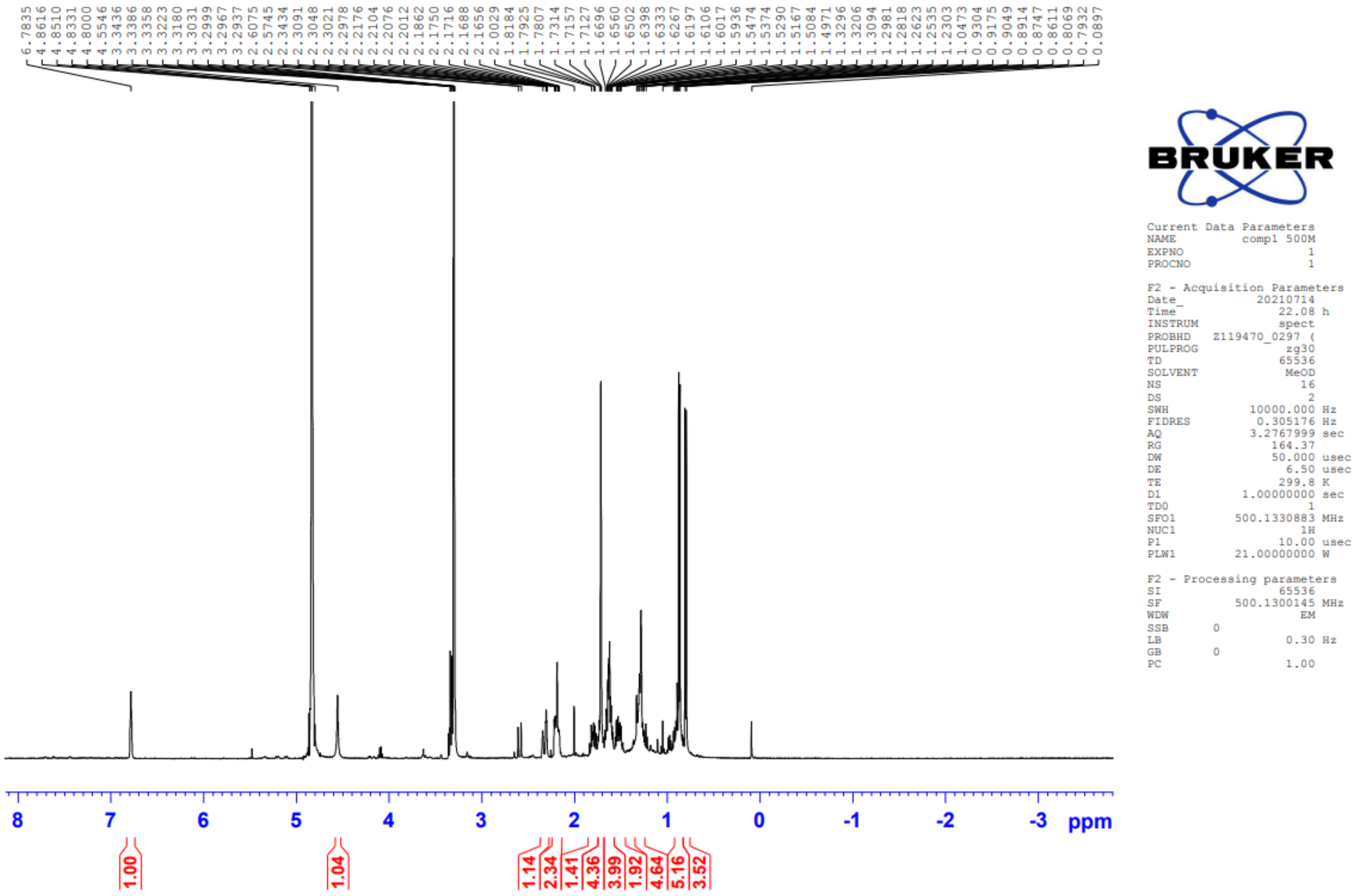

Figure S9. The NOESY spectrum of compound **1** (in CD_3OD, 500 MHz)

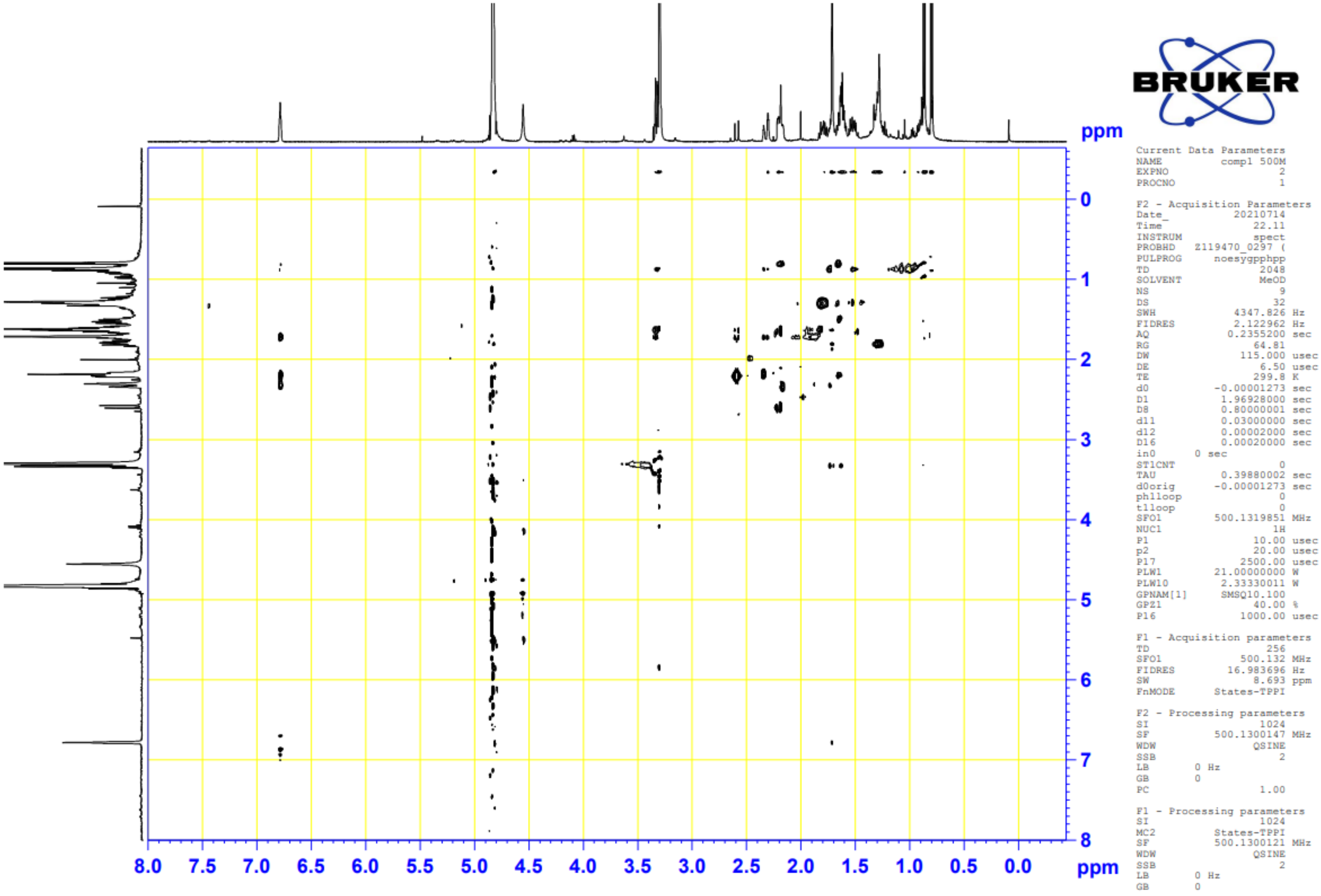

Figure S10. Structures of the co-isolated known compounds **2-11**

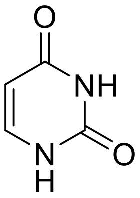

2

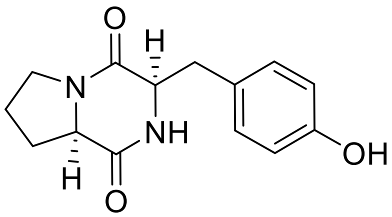

3

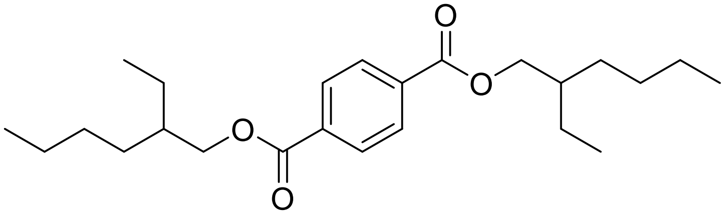

4

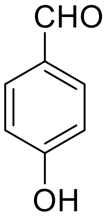

5

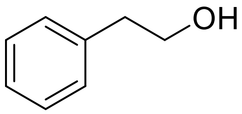

6

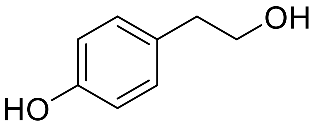

7

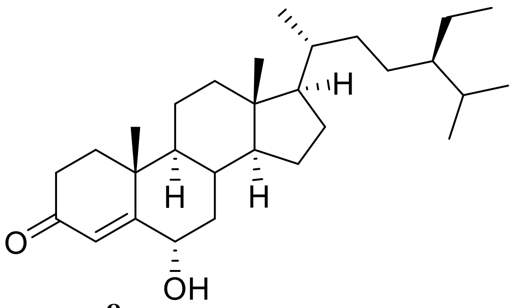

8

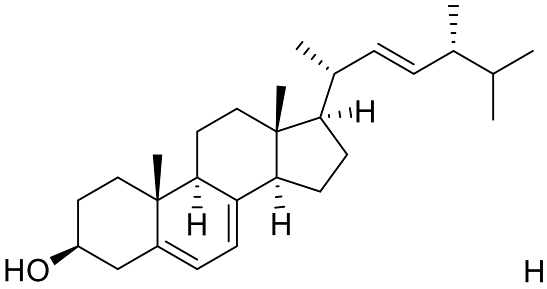

9

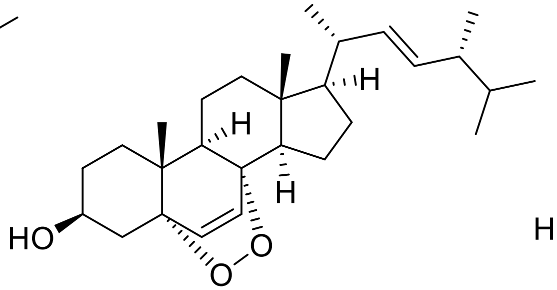

10

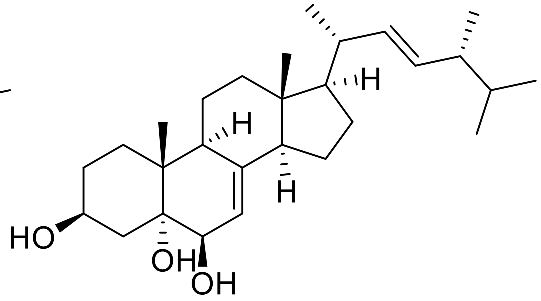

11

Figure S11. The ¹H-NMR spectrum of compound **2** (in DMSO, 600 MHz)

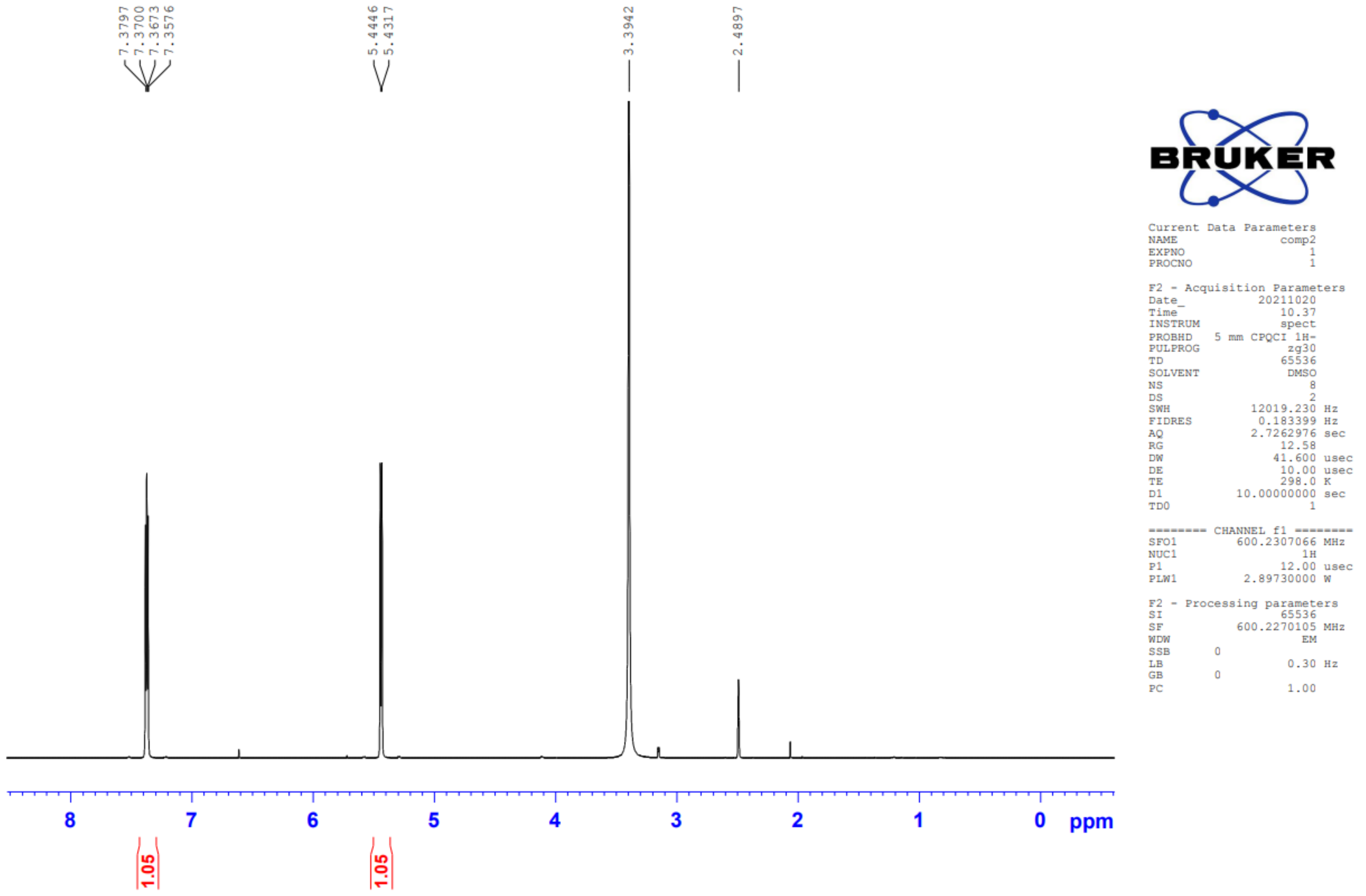

Figure S12. The ^{13}C-NMR spectrum of compound **2** (in DMSO, 150 MHz)

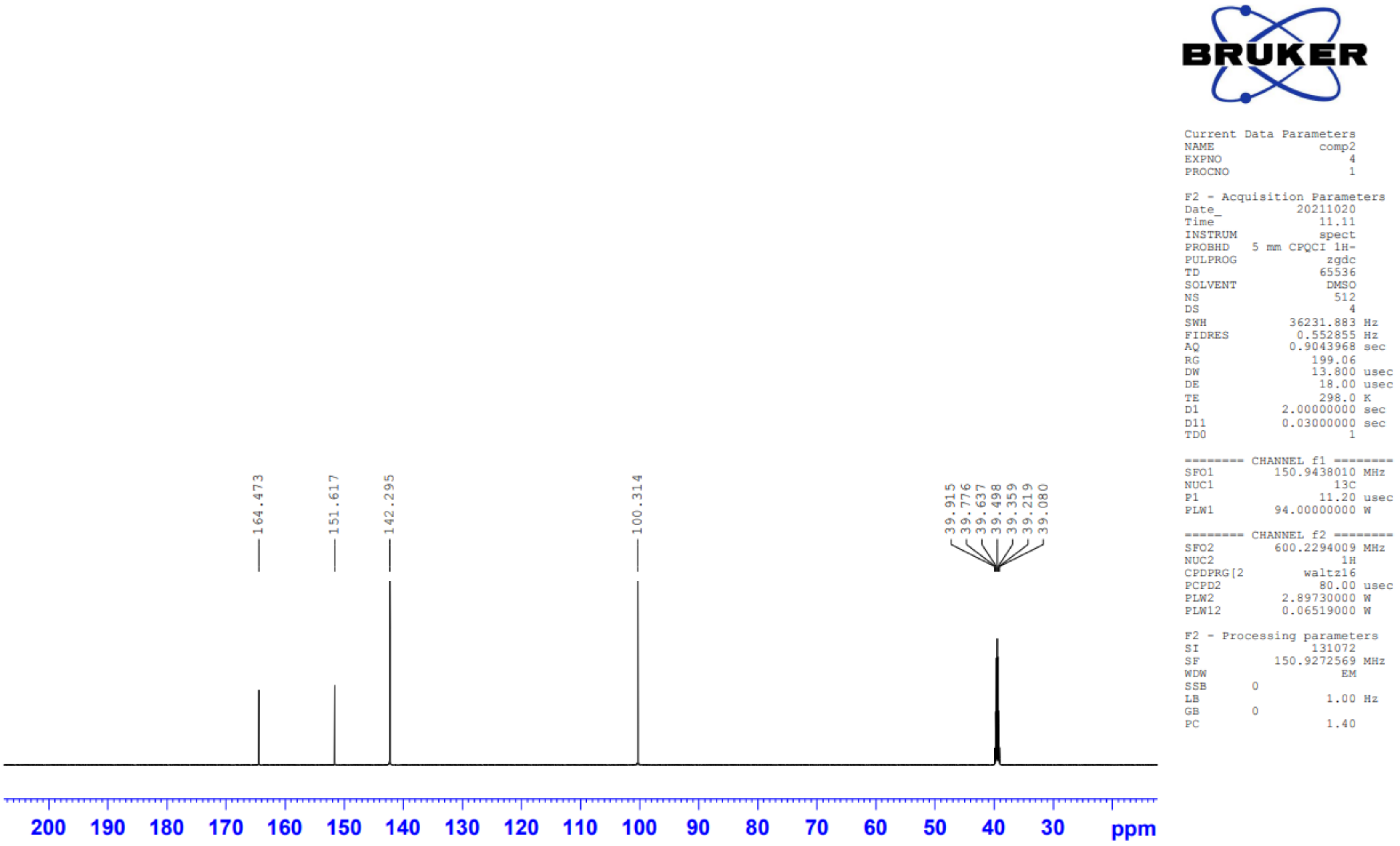

Figure S13. The ^{1}H-NMR spectrum of compound **3** (in $CDCl_3$, 400 MHz)

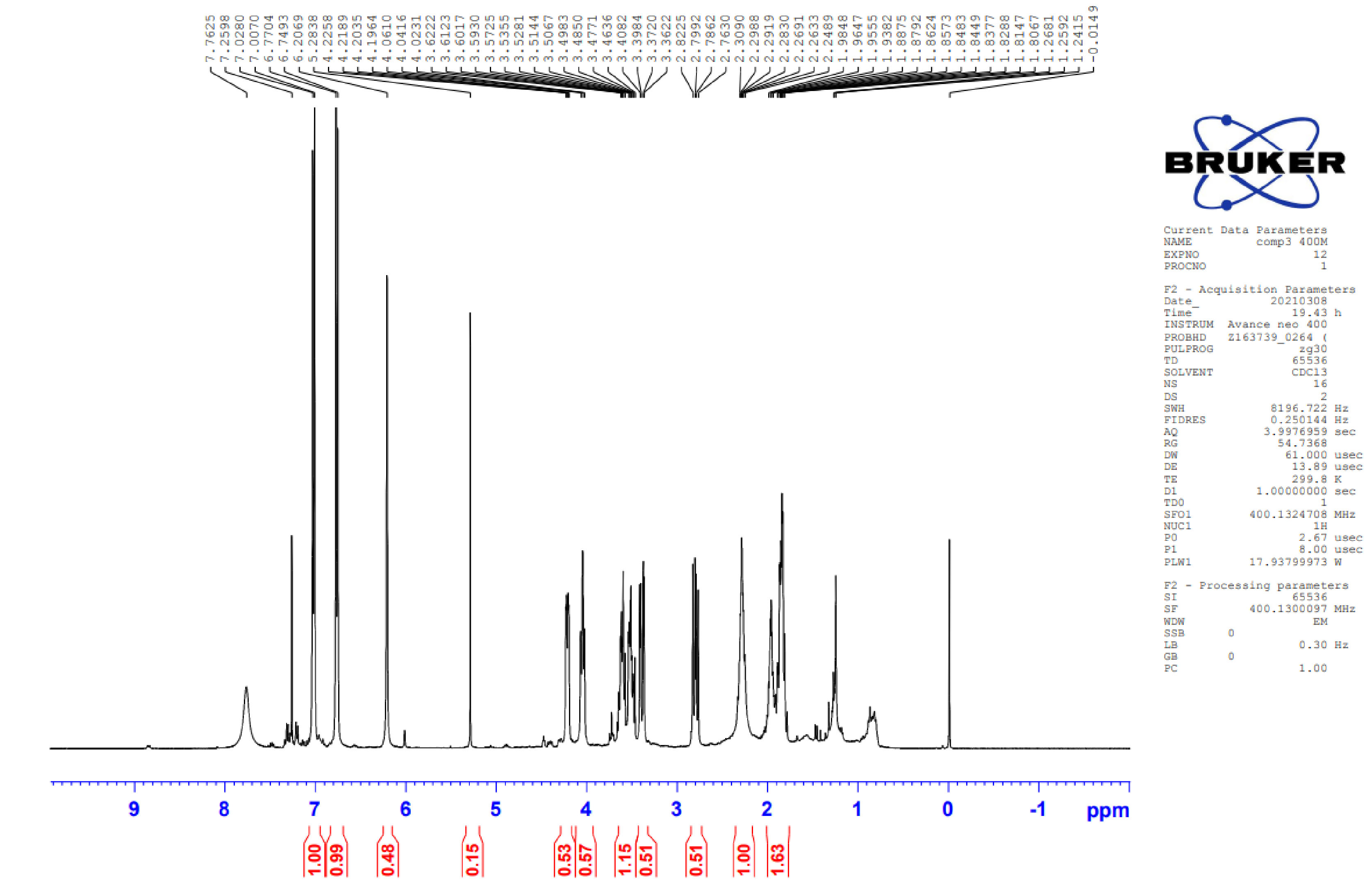

Figure S14. The ^{13}C-NMR spectrum of compound **3** (in $CDCl_3$, 100 MHz)

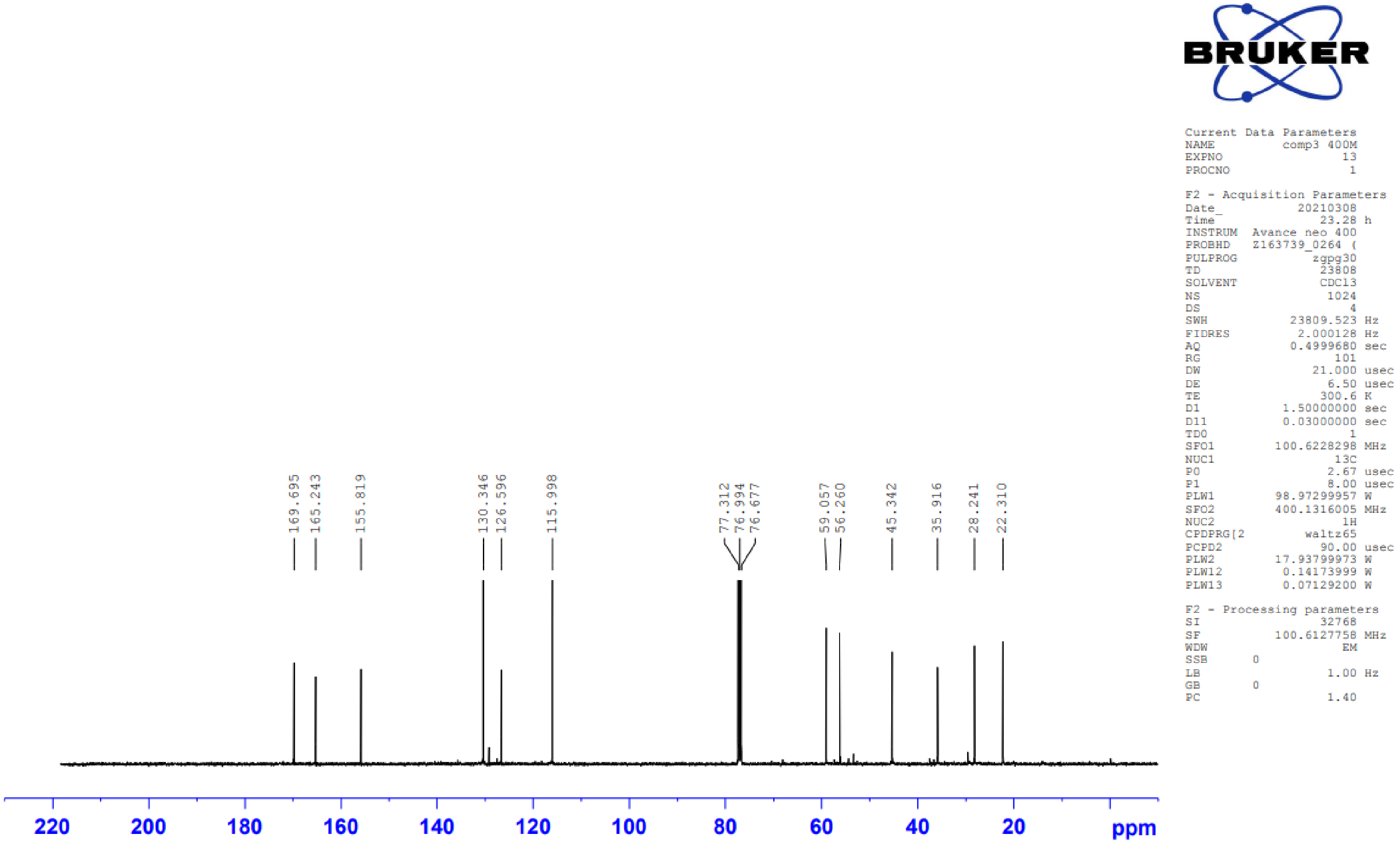

Figure S15. The ^{1}H-NMR spectrum of compound **4** (in CD3OD, 400 MHz)

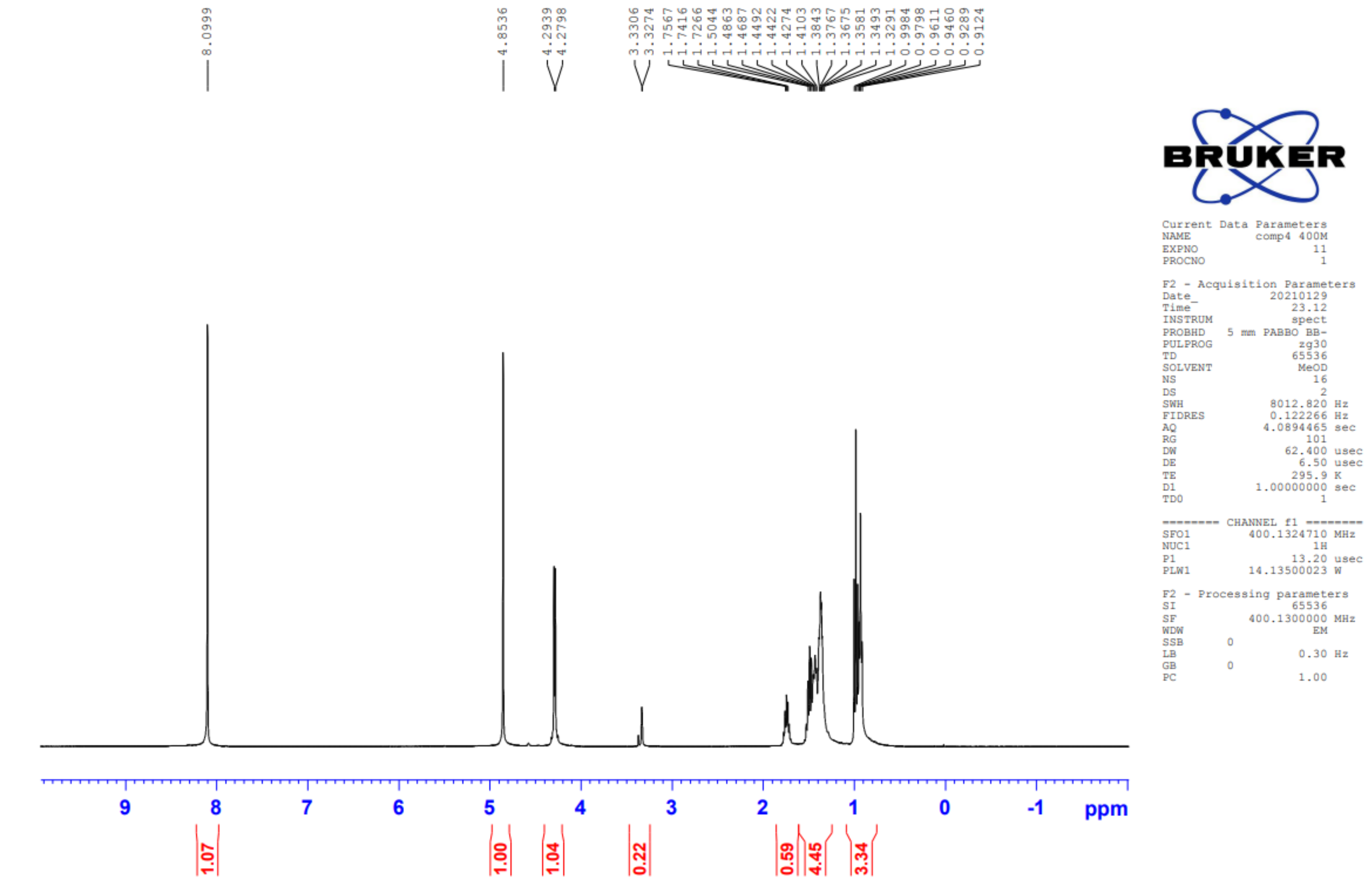

Figure S16. The ^{13}C-NMR spectrum of compound **4** (in CD3OD, 100 MHz)

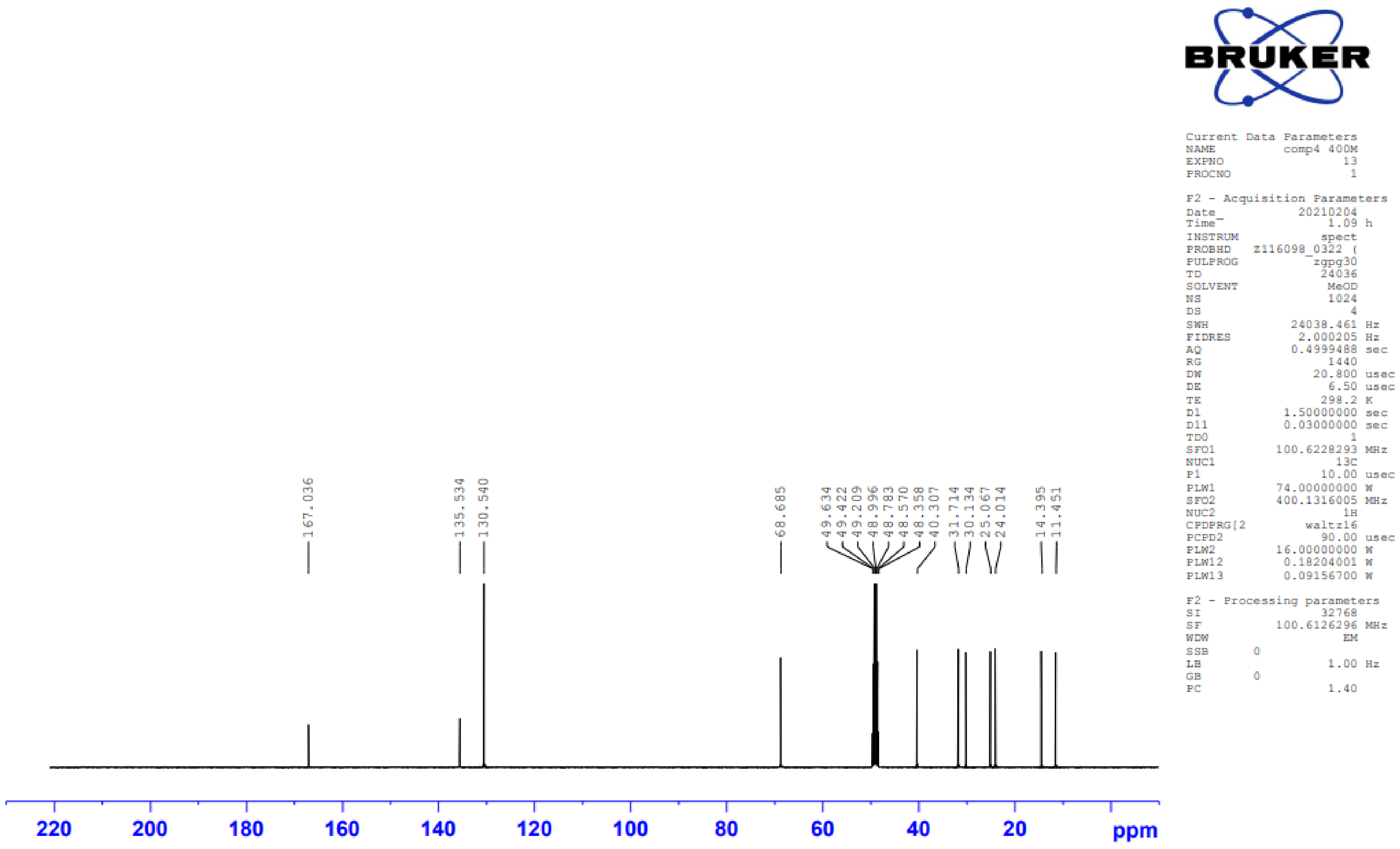

Figure S17. The ^{1}H-NMR spectrum of compound **5** (in CD3OD, 700 MHz)

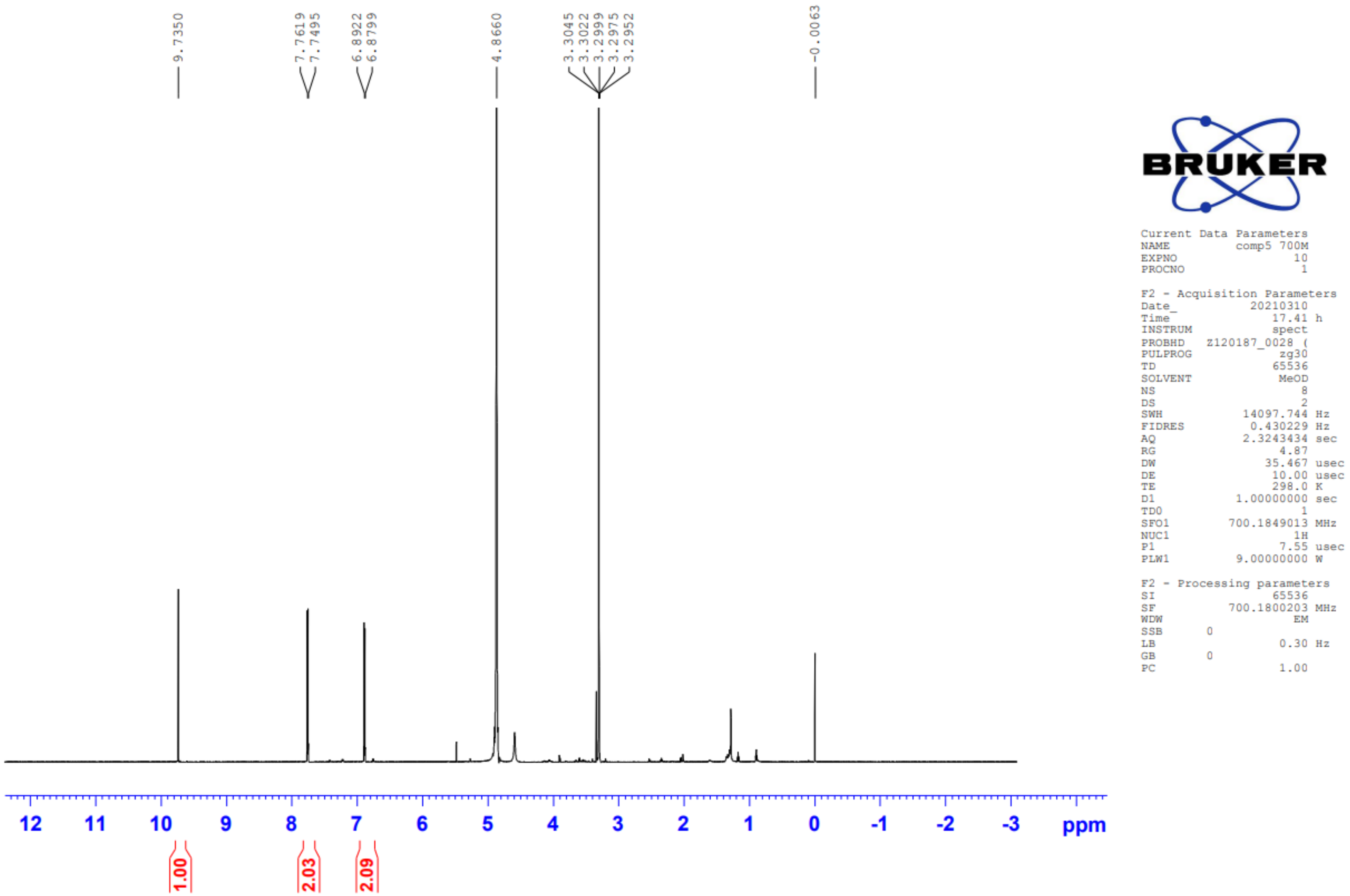

Figure S18. The ^{13}C-NMR spectrum of compound **5** (in CD3OD, 176 MHz)

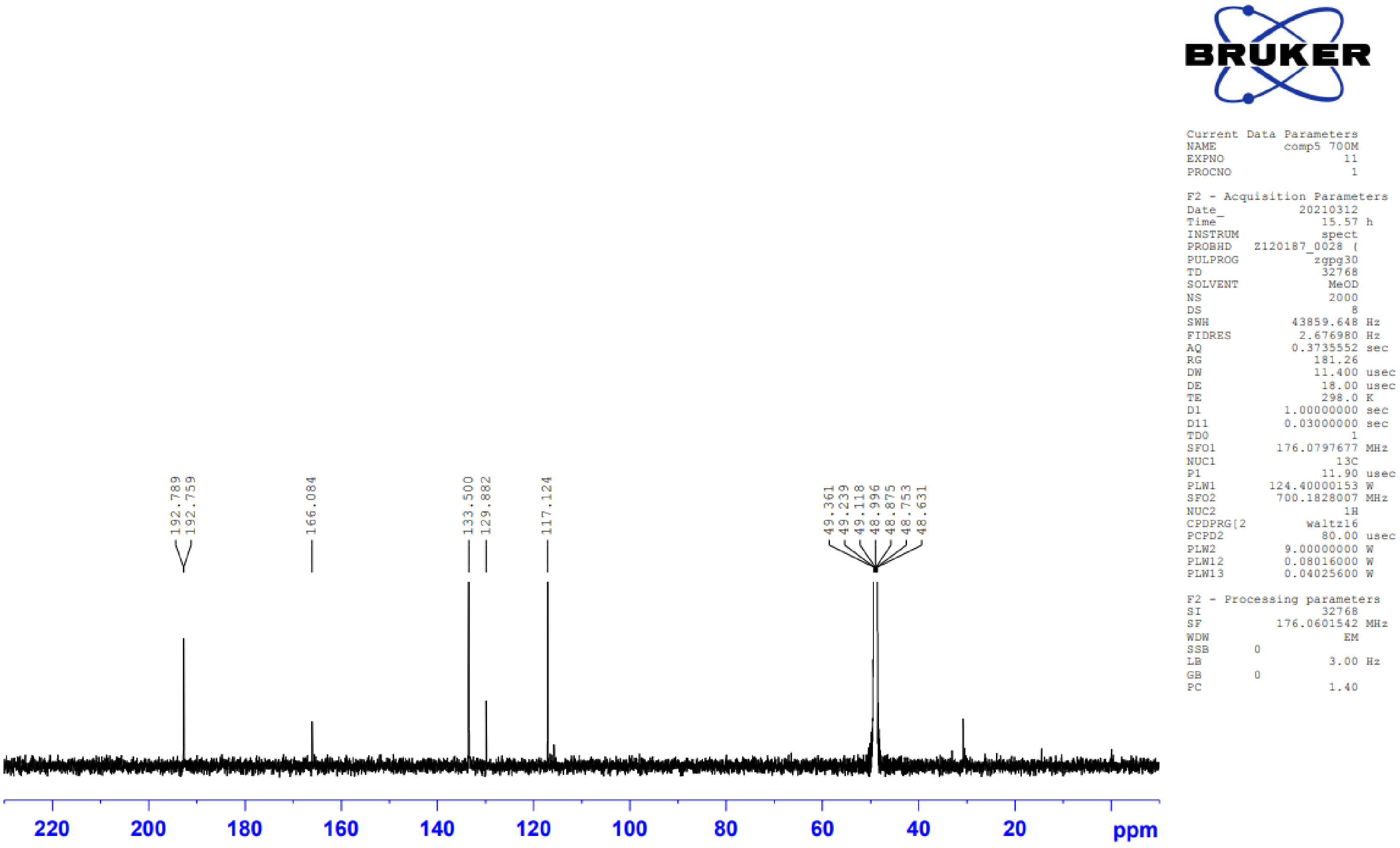

Figure S19. The ^{1}H-NMR spectrum of compound **6** (in $CDCl_3$, 600 MHz)

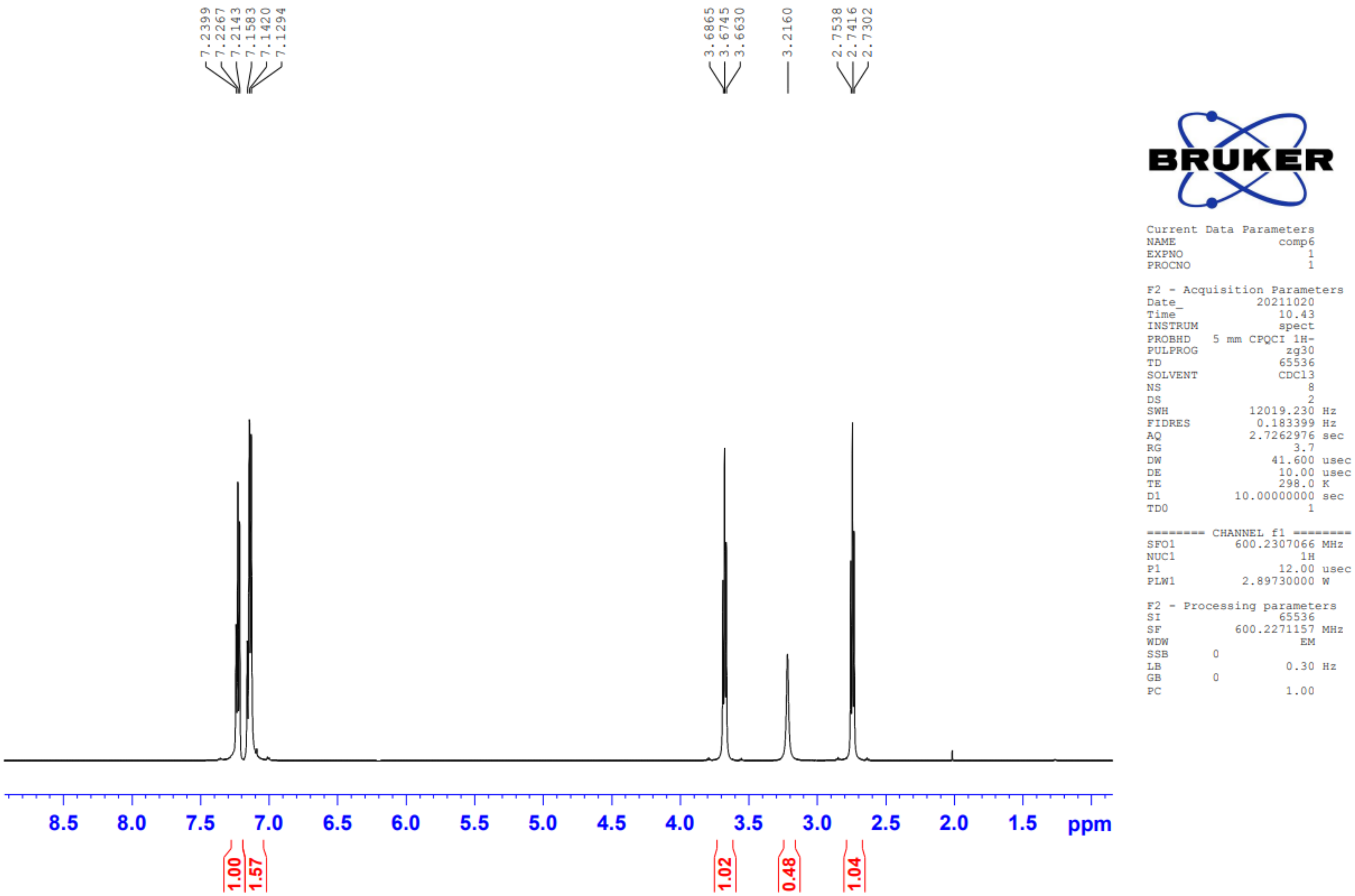

Figure S20. The ^{13}C-NMR spectrum of compound **6** (in $CDCl_3$, 150 MHz)

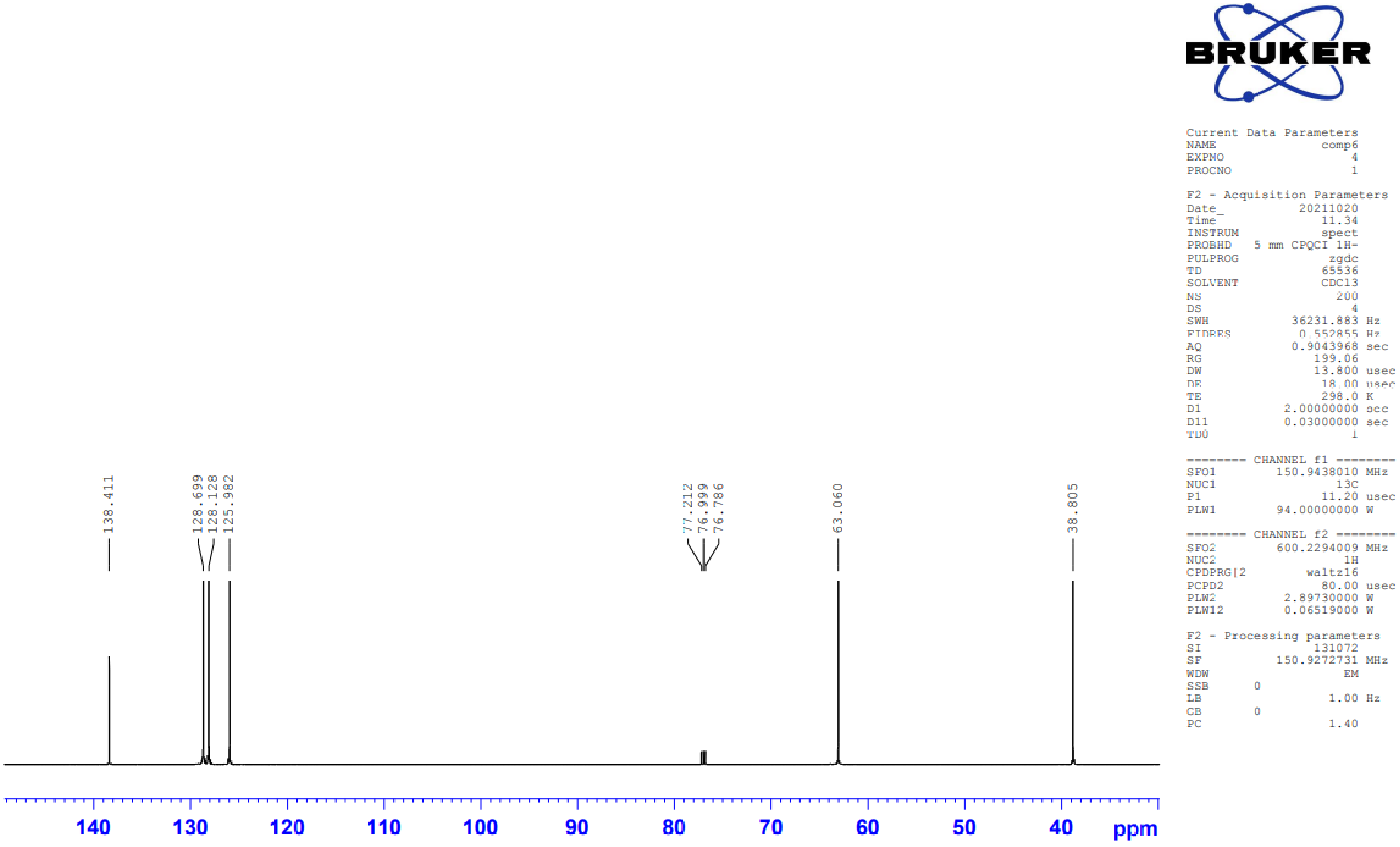

Figure S21. The ^{1}H-NMR spectrum of compound **7** (in CD3OD, 400 MHz)

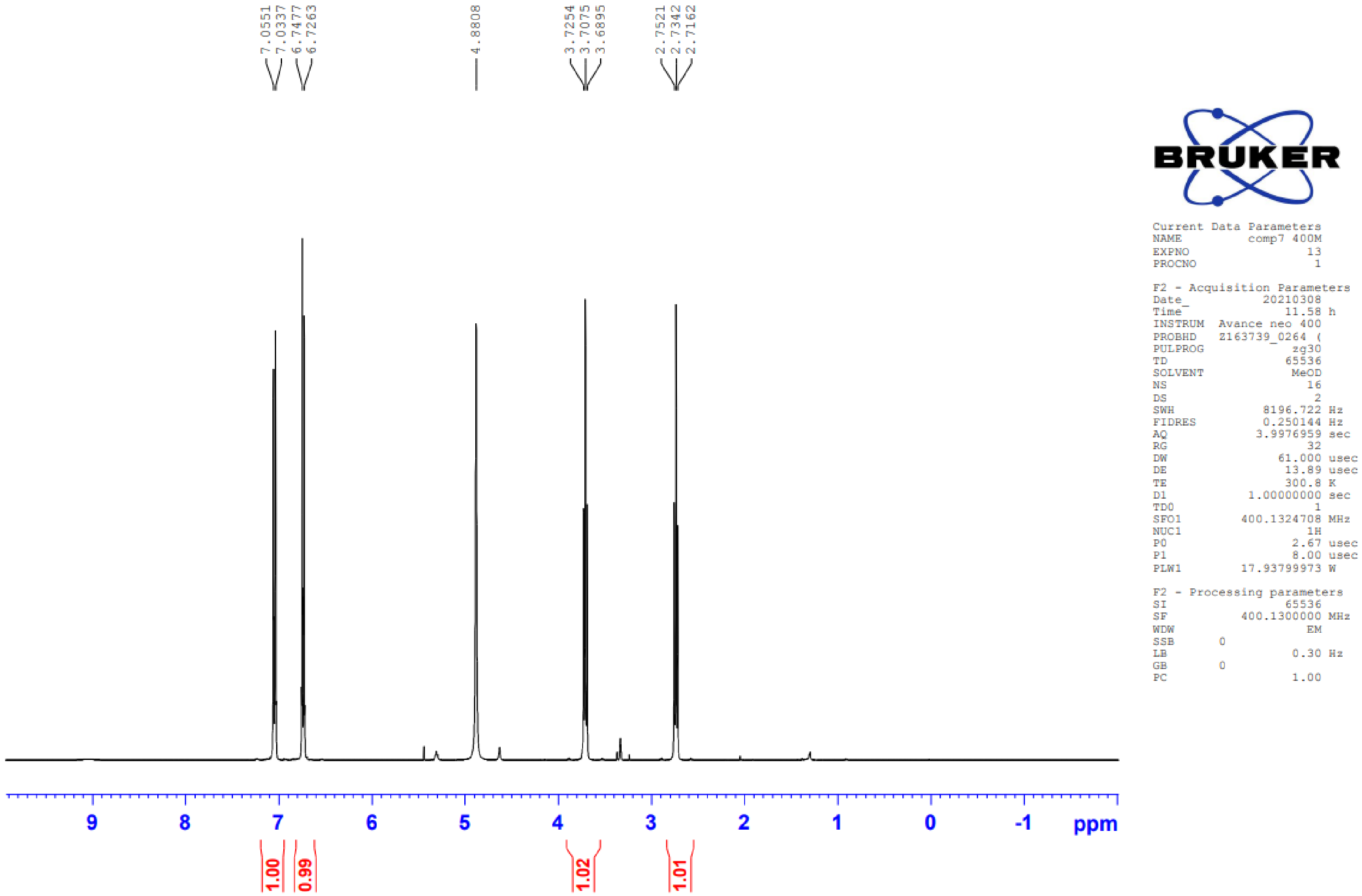

Figure S22. The ^{13}C-NMR spectrum of compound **7** (in CD3OD, 100 MHz)

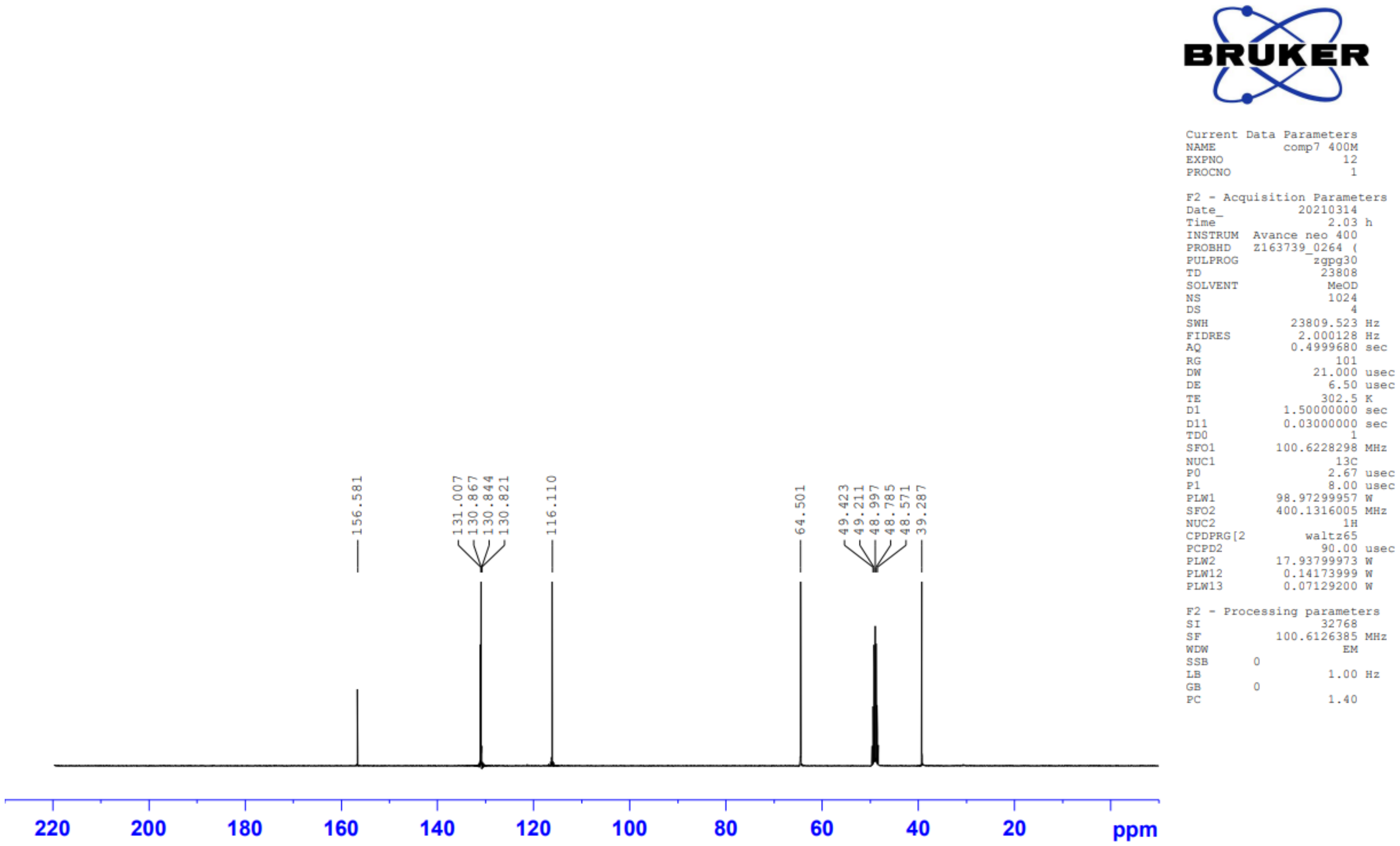

Figure S23. The ^{1}H-NMR spectrum of compound **8** (in $CDCl_3$, 400 MHz)

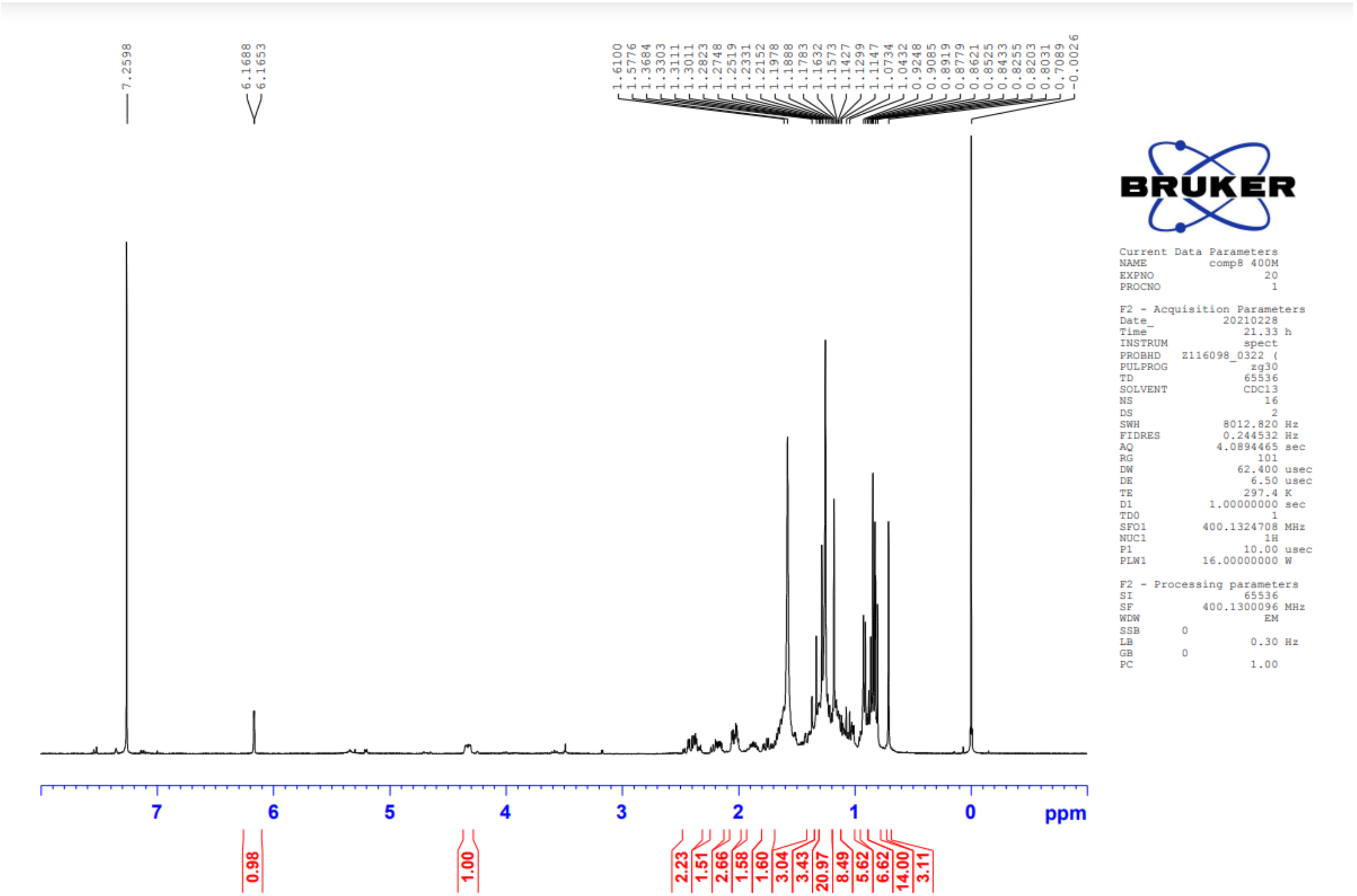

Figure S24. The ^{13}C-NMR spectrum of compound **8** (in $CDCl_3$, 100 MHz)

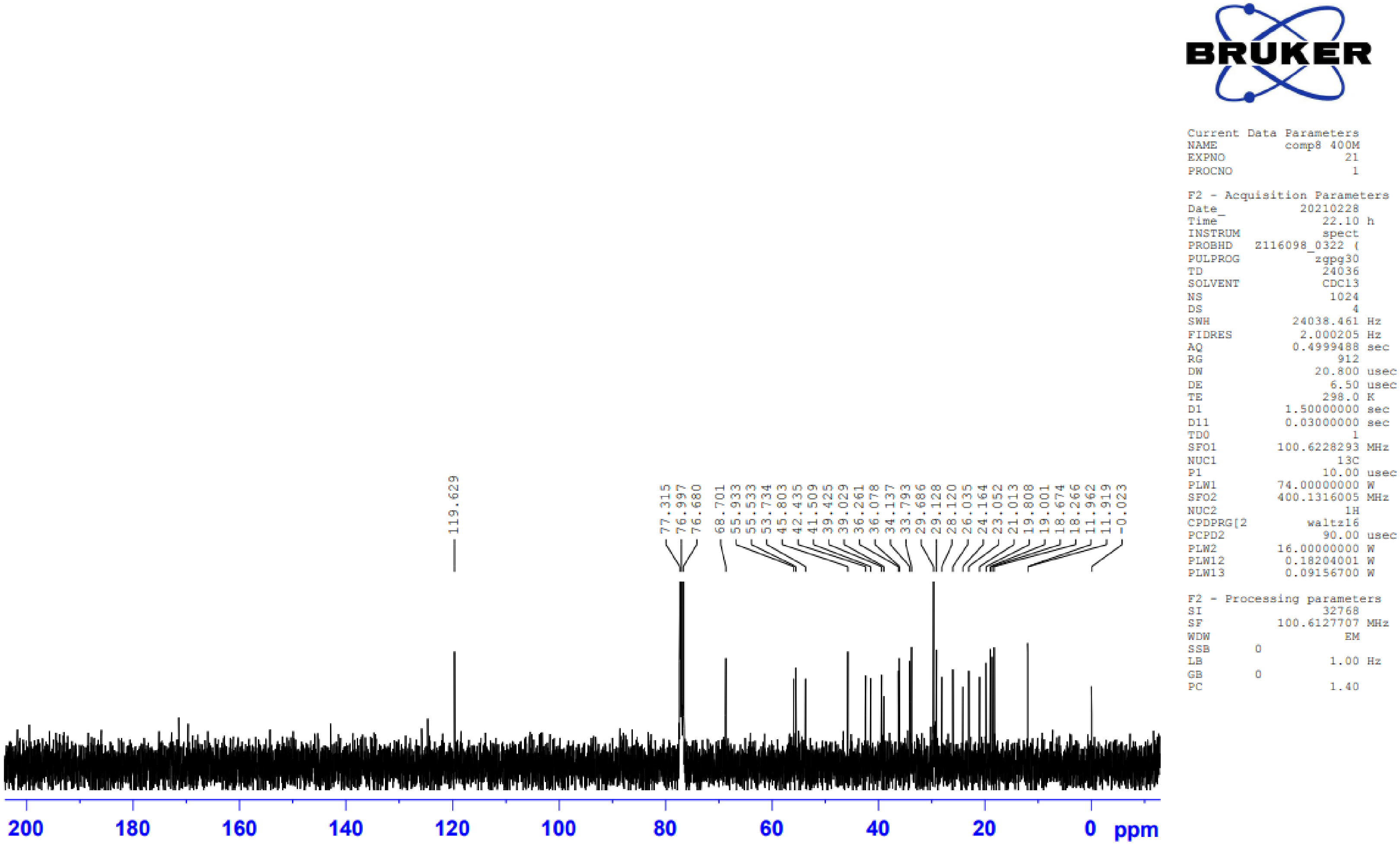

Figure S25. The ^{1}H-NMR spectrum of compound **9** (in $CDCl_3$, 400 MHz)

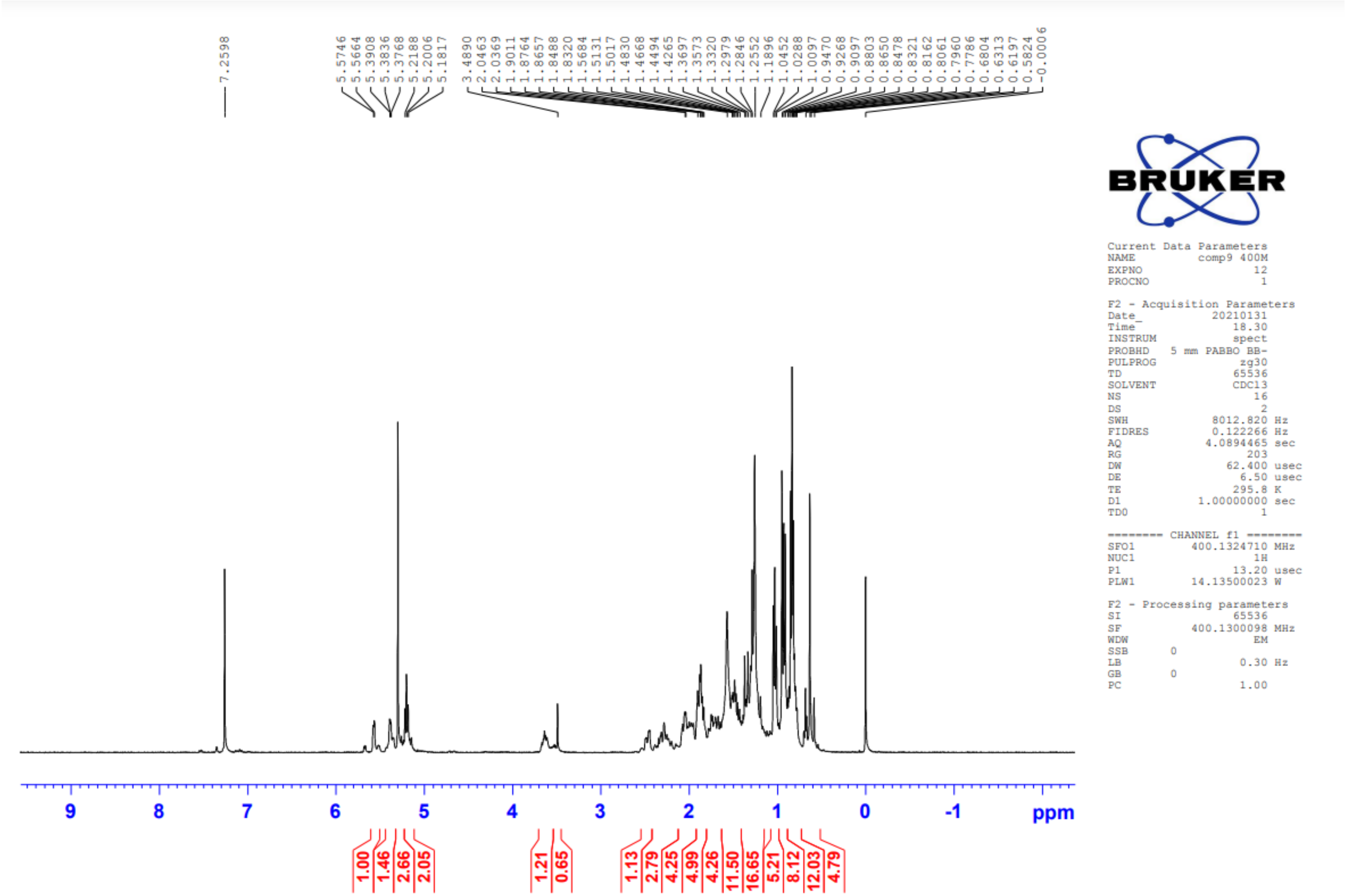

Figure S26. The ^{13}C-NMR spectrum of compound **9** (in $CDCl_3$, 100 MHz)

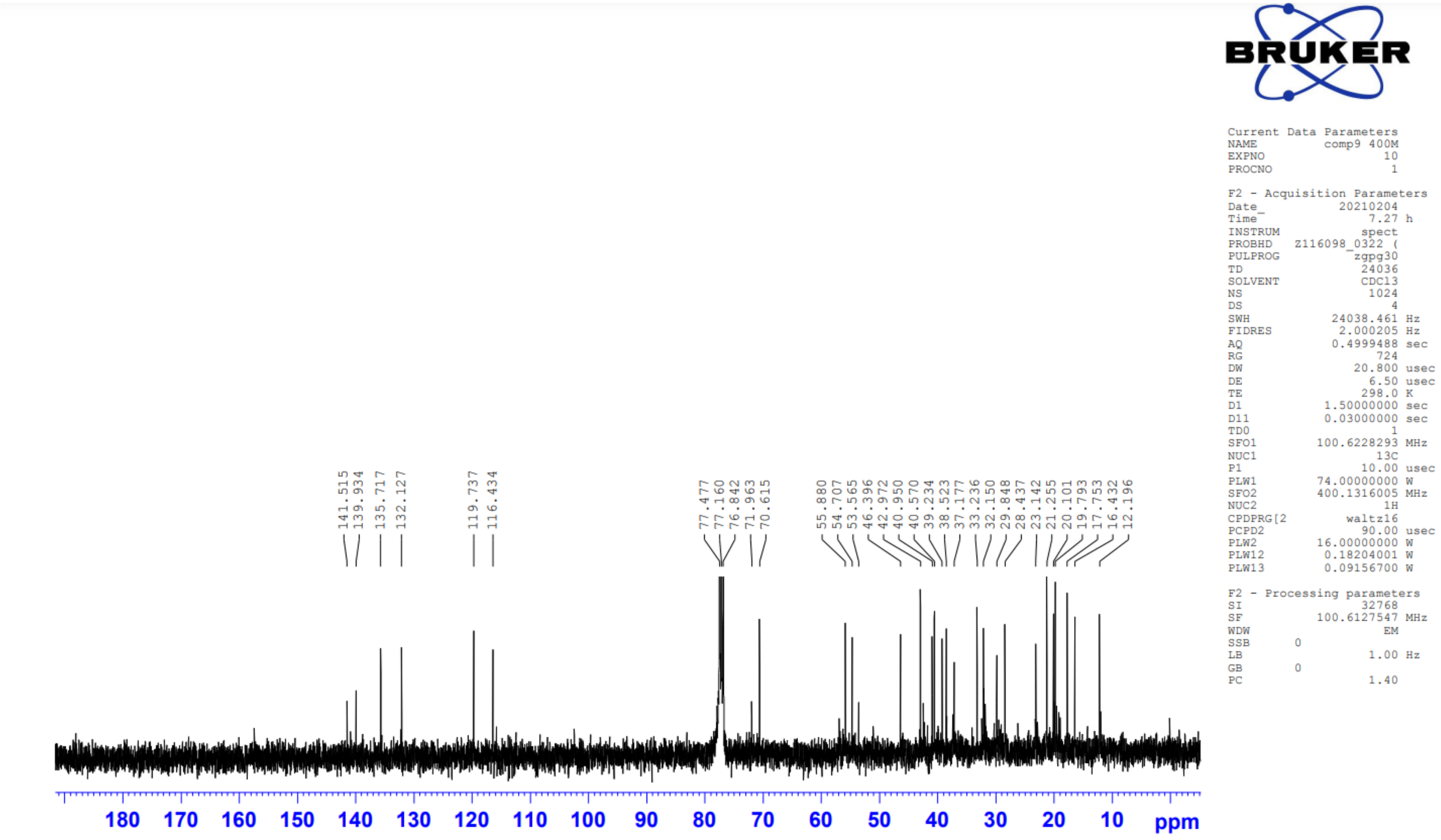

Figure S27. The ^{1}H-NMR spectrum of compound **10** (in $CDCl_3$, 400 MHz)

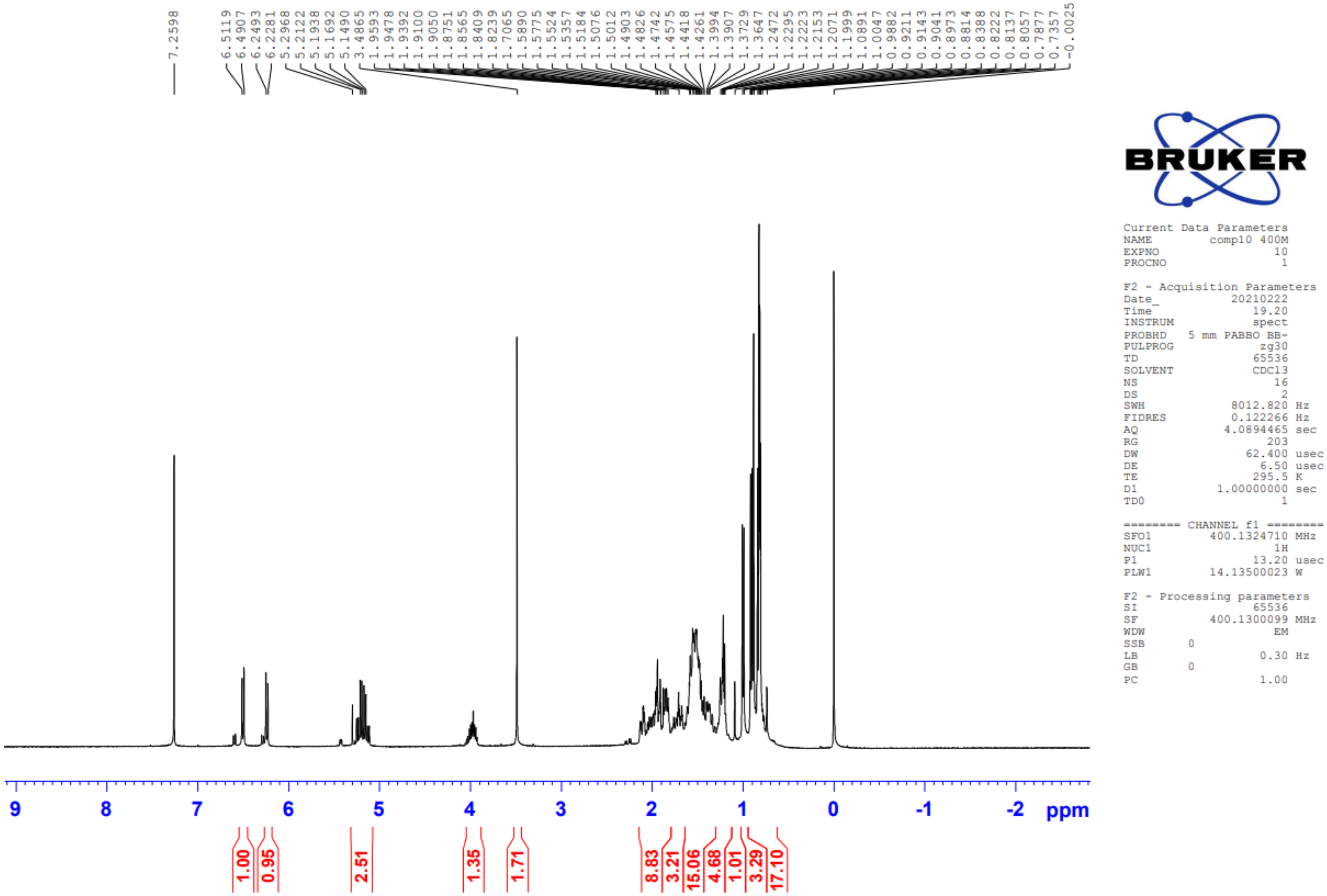

Figure S28. The ^{13}C-NMR spectrum of compound **10** (in $CDCl_3$, 100 MHz)

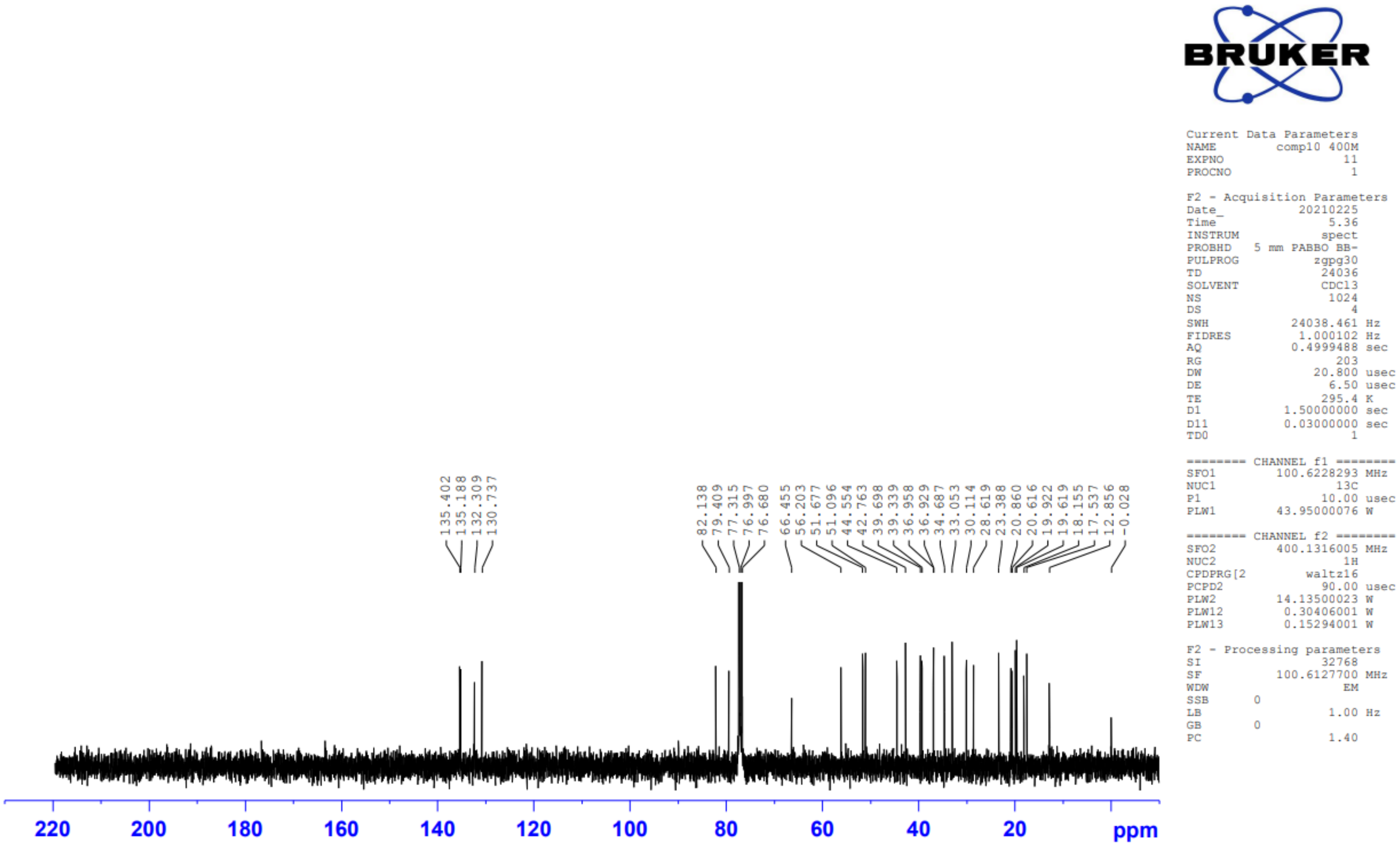

Figure S29. The ^{1}H-NMR spectrum of compound **11** (in DMSO, 700 MHz)

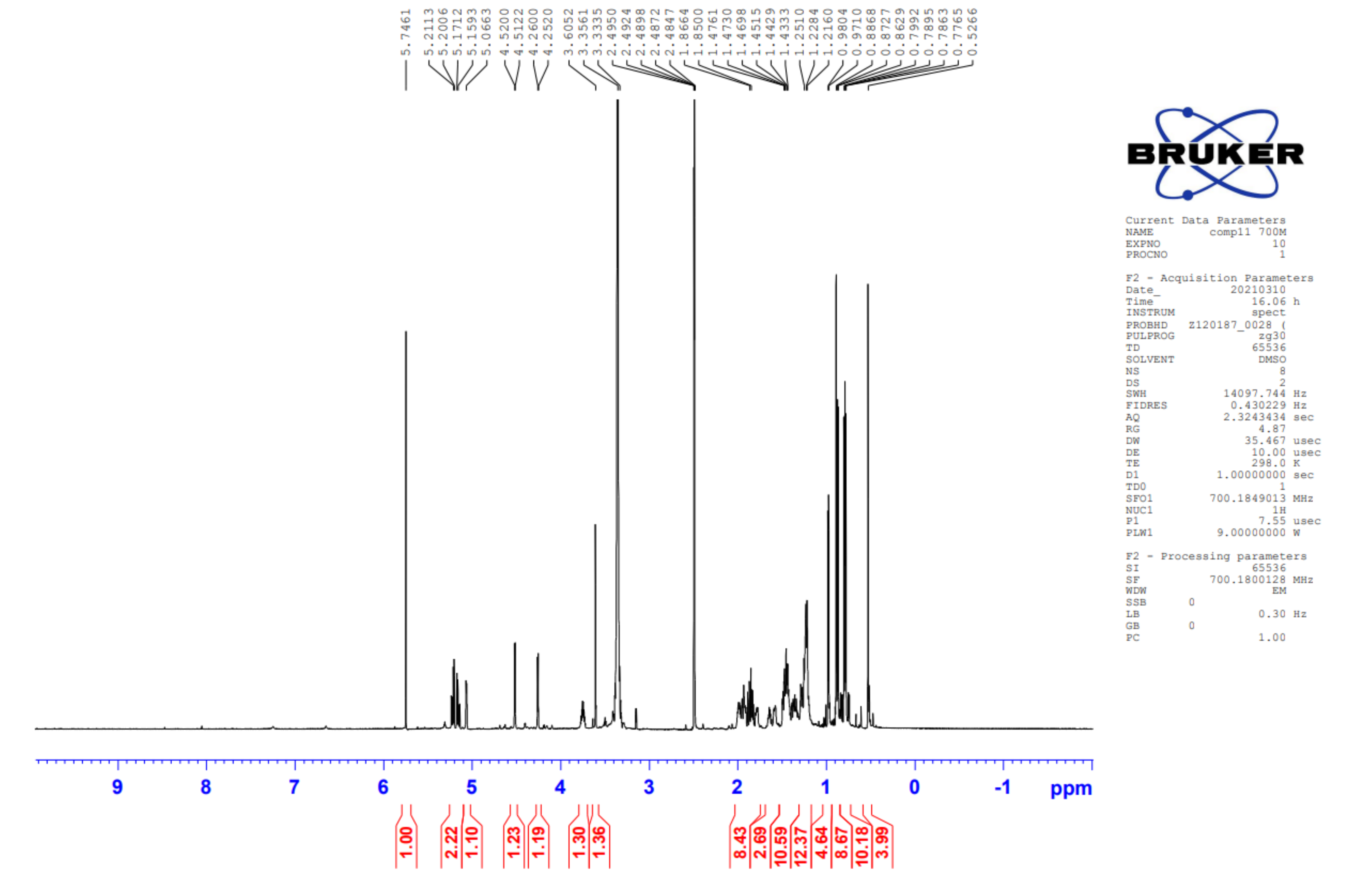

Figure S30. The ^{13}C-NMR spectrum of compound **11** (in DMSO, 176 MHz)

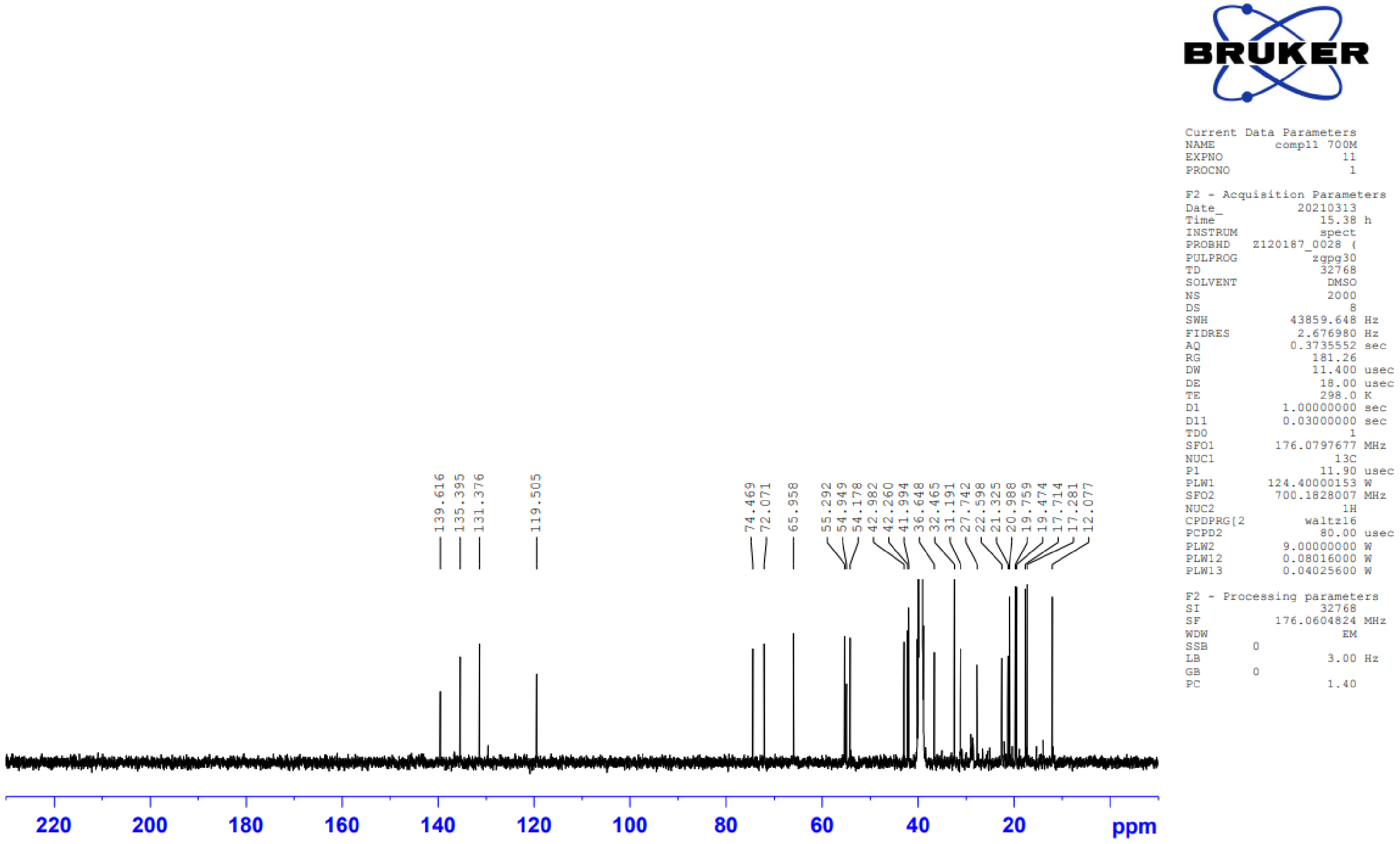

Figure S31. Optimized geometries of predominant conformers for compound **1a** at the B3LYP/6-31G (d,p) level in the gas phase

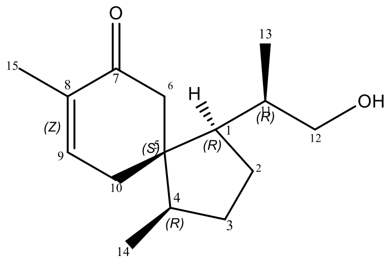

1a

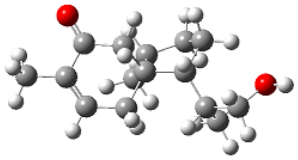

a

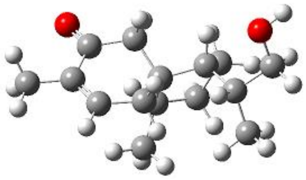

b

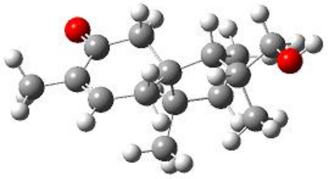

c

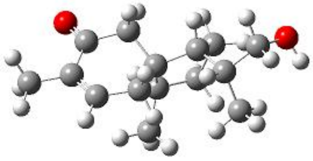

d

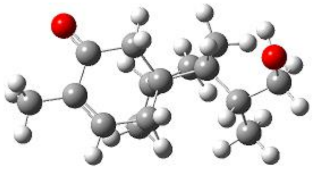

e

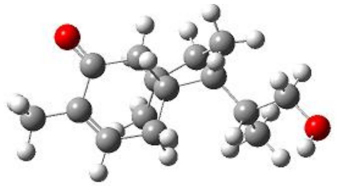

f

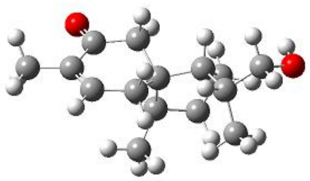

g

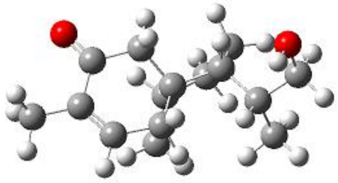

h

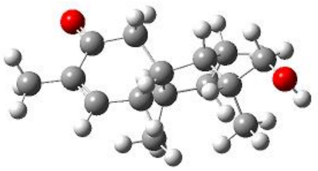

i

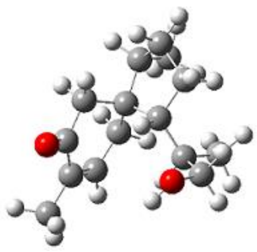

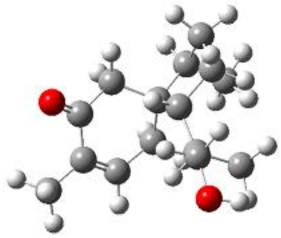

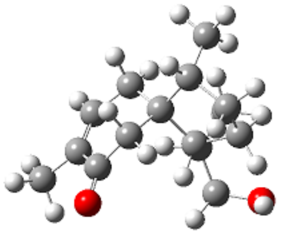

j **k** **l**

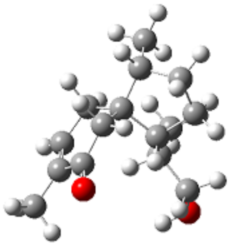

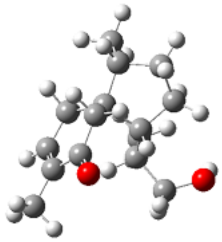

m **n**

Figure S32. Calculated and experimental ECDs of **1a** and **1b** (red, calculated at the B3LYP-PCM/6-31G (d,p)//B3LYP/6-31G (d,p) level in CH_3OH; blue, experimental in CH_3OH)

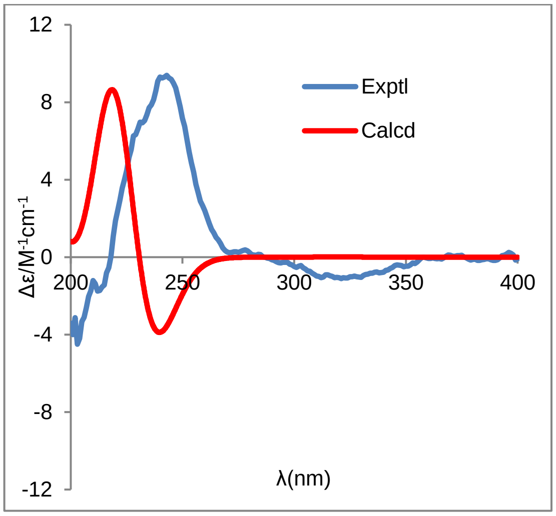

1a

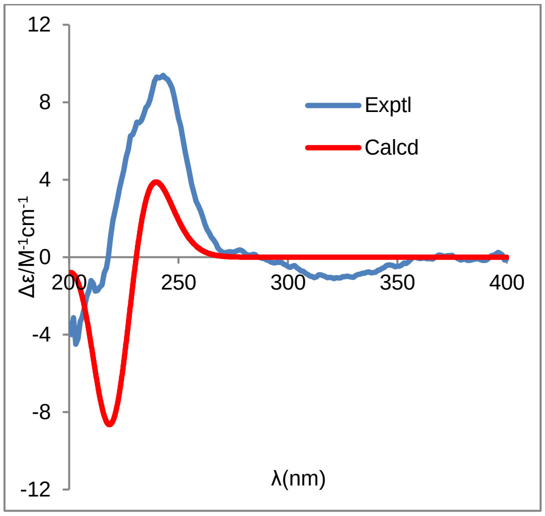

1b

Table S1. Important thermodynamic parameters (a. u.) and Boltzmann distributions of the optimized compound **1a** at B3LYP/6-31G (d,p) level in the gas phase

Conformations	E+ZPE	G	%
**1a-a**	-736.142187	-736.186302	0.9
**1a-b**	-736.143891	-736.188346	8
**1a-c**	-736.143040	-736.187407	3
**1a-d**	-736.142296	-736.186251	0.9
**1a-e**	-736.143587	-736.188025	5.7
**1a-f**	-736.143553	-736.187772	4.4
**1a-g**	-736.142003	-736.186013	0.7
**1a-h**	-736.144192	-736.188406	8.5
**1a-i**	-736.142976	-736.187277	2.6
**1a-j**	-736.145098	-736.189656	32.1
**1a-k**	-736.143654	-736.188193	6.8
**1a-l**	-736.143724	-736.187927	5.1
**1a-m**	-736.144563	-736.189033	16.6
**1a-n**	-736.189033	-736.187847	4.7

E+ZPE, G: total energy with zero point energy (ZPE) and Gibbs free energy in the gas phase at B3LYP/6-31G(d,p) level., %: Boltzmann distributions, using the relative Gibbs free energies as weighting factors

Table S2. Optimized Z-matrixes of compound **1a** in the gas phase (Å) at B3LYP/6-31G (d,p) level

**1a-a**				**1a-b**			
C	-3.0549	-0.56282	-0.46736	C	-2.92362	-0.6045	-0.58622
C	-2.13899	-0.64893	-1.45029	C	-1.9875	-0.50472	-1.54866
C	-0.64599	-0.6647	-1.2452	C	-0.50207	-0.42723	-1.30836
C	-0.18877	-0.07691	0.106829	C	-0.11669	0.028194	0.114887
C	-1.1033	-0.71848	1.184075	C	-0.99445	-0.80493	1.086579
C	-2.59003	-0.49898	0.943347	C	-2.48939	-0.6726	0.834033
C	-0.31834	1.504519	0.222569	C	-0.3725	1.568586	0.414062
C	1.02061	1.993745	0.834635	C	0.900185	2.074618	1.145504
C	1.704503	0.753857	1.426015	C	1.684669	0.824227	1.571771
C	1.305414	-0.41647	0.495677	C	1.390231	-0.24257	0.491525
O	-3.37468	-0.33103	1.8677	O	-3.30077	-0.6727	1.750739
C	-4.54036	-0.52413	-0.6982	C	-4.40338	-0.65423	-0.84984
H	1.28026	-1.34594	1.079954	H	1.440763	-1.23983	0.939746
C	2.30355	-0.72131	-0.662	C	2.436489	-0.30538	-0.65979
C	3.533239	-1.49484	-0.16048	C	3.692632	-1.05106	-0.18726
C	2.756405	0.468054	-1.52421	C	2.852293	1.028346	-1.298
O	4.378714	-0.63878	0.603981	O	3.333619	-2.3909	0.138934
C	-0.67805	2.288074	-1.04715	C	-0.74035	2.474447	-0.76871
H	-2.48338	-0.72479	-2.48203	H	-2.30764	-0.4868	-2.59075

H	-0.31314	-1.71291	-1.31367	H	-0.08093	-1.42857	-1.48998
H	-0.16552	-0.15653	-2.08735	H	-0.05136	0.216437	-2.07102
H	-0.94434	-1.80787	1.189873	H	-0.74639	-1.87083	0.970555
H	-0.87139	-0.35682	2.189484	H	-0.80955	-0.54642	2.13295
H	-1.11752	1.70815	0.945902	H	-1.21345	1.625497	1.115455
H	0.863706	2.781114	1.579612	H	0.652628	2.720919	1.994225
H	1.644968	2.430398	0.047592	H	1.500571	2.681682	0.459225
H	2.786481	0.855605	1.520853	H	2.754284	1.023549	1.699726
H	1.312212	0.560333	2.431888	H	1.323091	0.46779	2.543079
H	-4.78	-0.57626	-1.76357	H	-4.62007	-0.59424	-1.91969
H	-5.0371	-1.35343	-0.18338	H	-4.83994	-1.57676	-0.45241
H	-4.97082	0.39306	-0.28341	H	-4.91354	0.168656	-0.33877
H	1.803416	-1.43808	-1.32782	H	2.019164	-0.93804	-1.45275
H	3.203338	-2.35863	0.439063	H	4.443541	-1.03494	-0.99325
H	4.076899	-1.88981	-1.03449	H	4.135994	-0.5361	0.680358
H	3.394755	0.115955	-2.34249	H	3.554651	0.85447	-2.12067
H	1.912341	0.998171	-1.97166	H	1.997441	1.569456	-1.70998
H	3.341011	1.181749	-0.94015	H	3.348295	1.690259	-0.58013
H	5.146168	-1.15046	0.887718	H	4.094442	-2.81419	0.555064
H	-0.73312	3.356924	-0.81296	H	-0.88195	3.501447	-0.41436
H	-1.64692	1.99103	-1.45777	H	-1.66941	2.162816	-1.25395

H	0.07537	2.173472	-1.83408	H	0.047215	2.505268	-1.52923
**1a-c**				**1a-d**			
C	-2.9692	-0.89415	-0.28045	C	-3.05987	-0.62757	-0.3713
C	-1.96244	-1.29829	-1.078	C	-2.14309	-0.89191	-1.32096
C	-0.49733	-1.07366	-0.80743	C	-0.65052	-0.8768	-1.11244
C	-0.20658	0.071867	0.184863	C	-0.19358	-0.06581	0.118623
C	-1.17309	-0.1357	1.380895	C	-1.11289	-0.50829	1.288458
C	-2.64248	-0.18677	0.985078	C	-2.59746	-0.32147	1.008147
C	-0.44587	1.533896	-0.39381	C	-0.31684	1.511977	-0.03593
C	0.79306	2.366269	0.032046	C	1.012923	2.095878	0.512714
C	1.511686	1.553709	1.11872	C	1.696668	0.964276	1.292833
C	1.26476	0.069114	0.758055	C	1.298157	-0.34021	0.56219
O	-3.51754	0.284336	1.699606	O	-3.38218	0.013822	1.885459
C	-4.42602	-1.11047	-0.58509	C	-4.54461	-0.62018	-0.61072
H	1.2441	-0.52392	1.682169	H	1.26899	-1.16125	1.290214
C	2.383597	-0.58698	-0.10099	C	2.303946	-0.83205	-0.51966
C	3.597941	-0.89532	0.787715	C	3.546697	-1.49395	0.11584
C	2.83687	0.174824	-1.35464	C	2.745273	0.192076	-1.57716
O	4.512749	-1.6879	0.036069	O	4.441122	-0.60339	0.777703
C	-0.72587	1.67177	-1.89611	C	-0.64898	2.073673	-1.42485
H	-2.2037	-1.8426	-1.99123	H	-2.48598	-1.1465	-2.32401

H	-0.08604	-2.0125	-0.40403	H	-0.31991	-1.92172	-0.99813
H	0.022976	-0.92093	-1.75821	H	-0.16807	-0.52361	-2.02955
H	-0.94587	-1.09646	1.867929	H	-0.96142	-1.58144	1.481331
H	-1.06117	0.642066	2.141542	H	-0.87967	0.018667	2.217663
H	-1.32422	1.941912	0.120546	H	-1.12834	1.836445	0.626457
H	0.5132	3.367214	0.376926	H	0.842501	2.983913	1.130462
H	1.451583	2.506249	-0.83139	H	1.64317	2.419075	-0.32338
H	2.574937	1.803955	1.194352	H	2.77899	1.077571	1.375149
H	1.073107	1.768938	2.099854	H	1.306335	0.934068	2.316933
H	-4.56062	-1.63522	-1.53456	H	-4.78159	-0.8575	-1.65117
H	-4.90912	-1.68901	0.20956	H	-5.04867	-1.34249	0.040143
H	-4.95668	-0.15405	-0.63114	H	-4.96936	0.358616	-0.36548
H	2.019128	-1.56981	-0.42593	H	1.815659	-1.65804	-1.05556
H	4.071798	0.042307	1.120575	H	3.233556	-2.21297	0.88008
H	3.265696	-1.43355	1.690094	H	4.079868	-2.06159	-0.66389
H	3.609627	-0.40062	-1.86925	H	3.406635	-0.282	-2.31265
H	2.015374	0.345015	-2.0552	H	1.900829	0.613502	-2.12643
H	3.267637	1.149383	-1.09969	H	3.290008	1.030318	-1.13088
H	5.315911	-1.79642	0.559943	H	4.887075	-0.0759	0.10301
H	-0.86894	2.728941	-2.14554	H	-0.69801	3.166964	-1.37351
H	-1.6307	1.13768	-2.19863	H	-1.61411	1.719398	-1.79747

H	0.105983	1.30914	-2.50926	H	0.115018	1.825113	-2.16937
**1a-e**				**1a-f**			
C	-2.93867	-0.55576	-0.61688	C	2.973504	-0.89003	0.278173
C	-2.01084	-0.38194	-1.57693	C	1.968224	-1.30122	1.073764
C	-0.52281	-0.33066	-1.34603	C	0.502306	-1.08122	0.802381
C	-0.12309	0.009888	0.105249	C	0.208257	0.069079	-0.1832
C	-0.99467	-0.89539	1.016023	C	1.175024	-0.12912	-1.38057
C	-2.49219	-0.73901	0.788881	C	2.6446	-0.17882	-0.985
C	-0.36805	1.521593	0.53068	C	0.44454	1.527649	0.404585
C	0.916729	1.964515	1.2818	C	-0.79051	2.363314	-0.02691
C	1.700482	0.681948	1.601424	C	-1.51001	1.551918	-1.11414
C	1.388242	-0.29349	0.442333	C	-1.26318	0.066949	-0.75537
O	-3.29354	-0.81126	1.711157	O	3.518503	0.296789	-1.6976
C	-4.42095	-0.57785	-0.87023	C	4.431047	-1.1014	0.582513
H	1.420495	-1.32676	0.809035	H	-1.24367	-0.52482	-1.6807
C	2.423105	-0.27814	-0.71771	C	-2.38635	-0.58663	0.100151
C	3.725801	-0.99343	-0.29909	C	-3.60096	-0.89517	-0.80091
C	2.788103	1.094041	-1.30283	C	-2.8482	0.175288	1.349447
O	3.543142	-2.35439	0.076808	O	-4.63779	-1.6012	-0.12908
C	-0.75035	2.520411	-0.56933	C	0.711482	1.656706	1.910045
H	-2.34033	-0.27862	-2.61099	H	2.211117	-1.84776	1.985326

H	-0.10819	-1.31605	-1.61049	H	0.09629	-2.01955	0.391617
H	-0.07543	0.368651	-2.05944	H	-0.02015	-0.93619	1.753363
H	-0.7535	-1.95001	0.812942	H	0.95011	-1.08723	-1.87397
H	-0.79973	-0.72138	2.078119	H	1.061535	0.65294	-2.13646
H	-1.19787	1.522994	1.247471	H	1.327918	1.937153	-0.09972
H	0.684368	2.543856	2.18144	H	-0.50644	3.362442	-0.37324
H	1.511772	2.619772	0.636534	H	-1.45046	2.507785	0.834659
H	2.772138	0.868712	1.730414	H	-2.57287	1.803167	-1.18931
H	1.34617	0.254188	2.546462	H	-1.0722	1.767339	-2.0954
H	-4.64617	-0.42981	-1.92962	H	4.567655	-1.63049	1.529252
H	-4.85839	-1.52796	-0.54555	H	4.917077	-1.67323	-0.21514
H	-4.92285	0.202735	-0.28946	H	4.957174	-0.14275	0.633811
H	2.011	-0.89156	-1.52841	H	-2.01039	-1.56692	0.431247
H	4.405136	-1.01037	-1.15852	H	-4.06424	0.034373	-1.14863
H	4.234861	-0.42762	0.49667	H	-3.26711	-1.44588	-1.69496
H	3.490099	0.976073	-2.13589	H	-3.64638	-0.38491	1.842783
H	1.915223	1.626777	-1.68688	H	-2.03958	0.325952	2.069154
H	3.270654	1.739064	-0.56087	H	-3.25733	1.157639	1.091171
H	3.264159	-2.37628	0.999848	H	-4.28275	-2.46184	0.130852
H	-0.8901	3.513956	-0.12935	H	0.852437	2.712361	2.166714
H	-1.68417	2.247546	-1.06838	H	1.613689	1.120911	2.217522

H	0.028722	2.616847	-1.333	H	-0.12591	1.290949	2.513771
**1a-g**				**1a-h**			
C	-3.05012	-0.44982	-0.58065	C	-2.92933	-0.59137	-0.59001
C	-2.14277	-0.29589	-1.56331	C	-1.99626	-0.48048	-1.55395
C	-0.64774	-0.34282	-1.37841	C	-0.50969	-0.41186	-1.31634
C	-0.18515	-0.08645	0.070354	C	-0.11856	0.023276	0.111733
C	-1.08288	-0.97282	0.974353	C	-0.99561	-0.81974	1.075526
C	-2.57399	-0.71672	0.801833	C	-2.49117	-0.68242	0.828064
C	-0.33114	1.420345	0.556432	C	-0.37032	1.560313	0.430004
C	0.992144	1.757606	1.293421	C	0.903544	2.053309	1.168595
C	1.707423	0.421929	1.543325	C	1.687224	0.796018	1.575708
C	1.316614	-0.49078	0.353275	C	1.38901	-0.2545	0.480758
O	-3.35057	-0.77935	1.745604	O	-3.30038	-0.69694	1.746211
C	-4.53787	-0.3711	-0.78634	C	-4.41005	-0.63138	-0.84966
H	1.299745	-1.5365	0.691543	H	1.442521	-1.25764	0.917262
C	2.316832	-0.48555	-0.84178	C	2.436191	-0.3025	-0.66852
C	3.518123	-1.41755	-0.57116	C	3.704968	-1.04286	-0.19651
C	2.817401	0.88649	-1.31674	C	2.8472	1.036209	-1.2996
O	4.407377	-0.98025	0.453704	O	3.463152	-2.36623	0.267364
C	-0.67239	2.481615	-0.49811	C	-0.73406	2.481932	-0.74178
H	-2.49577	-0.13237	-2.58166	H	-2.31942	-0.44498	-2.59476

H	-0.3041	-1.33989	-1.69748	H	-0.09375	-1.41296	-1.51322
H	-0.17992	0.357913	-2.07747	H	-0.05785	0.23965	-2.07165
H	-0.91495	-2.03178	0.724999	H	-0.74999	-1.88506	0.947899
H	-0.84573	-0.85311	2.03531	H	-0.80763	-0.57412	2.124301
H	-1.14554	1.443764	1.29032	H	-1.21194	1.610914	1.130984
H	0.813193	2.311785	2.220633	H	0.657245	2.686421	2.027419
H	1.611914	2.402158	0.661425	H	1.503894	2.67057	0.491335
H	2.787893	0.548213	1.644106	H	2.757044	0.991158	1.704758
H	1.346013	-0.02449	2.477387	H	1.327568	0.425004	2.541945
H	-4.78533	-0.17384	-1.83264	H	-4.62986	-0.5543	-1.91777
H	-5.02458	-1.30219	-0.47723	H	-4.84906	-1.55785	-0.46445
H	-4.97124	0.419663	-0.1655	H	-4.91516	0.185744	-0.32459
H	1.806958	-0.96304	-1.68985	H	2.015322	-0.92381	-1.47344
H	3.153714	-2.43547	-0.36148	H	4.44517	-1.04316	-1.01234
H	4.12879	-1.47497	-1.47927	H	4.162139	-0.51673	0.648576
H	3.44524	0.766946	-2.20661	H	3.562002	0.871119	-2.11361
H	1.998458	1.559527	-1.58175	H	1.993905	1.573367	-1.72006
H	3.430194	1.37293	-0.55351	H	3.330155	1.697134	-0.57254
H	3.999114	-1.18013	1.304795	H	3.138741	-2.88358	-0.4818
H	-0.73414	3.465047	-0.01936	H	-0.87248	3.504638	-0.37421
H	-1.63425	2.291237	-0.98199	H	-1.66381	2.180029	-1.23194

H	0.092987	2.554566	-1.27809	H	0.054605	2.520071	-1.50078
**1a-i**				**1a-j**			
C	2.977949	-0.88442	0.288941	C	-2.4689	-0.49952	0.593135
C	1.973239	-1.289	1.088727	C	-1.58772	-0.67964	1.594874
C	0.506922	-1.07373	0.81628	C	-0.1723	-1.16582	1.425887
C	0.210547	0.06375	-0.18345	C	0.397138	-1.00135	-0.00129
C	1.178453	-0.14649	-1.37794	C	-0.707	-1.51599	-0.9641
C	2.647777	-0.18533	-0.98045	C	-2.04904	-0.81821	-0.79752
C	0.441696	1.53082	0.385853	C	1.691974	-1.84967	-0.24926
C	-0.7982	2.355986	-0.05231	C	2.355025	-1.13593	-1.44456
C	-1.51161	1.530137	-1.13253	C	2.177964	0.35818	-1.13199
C	-1.26074	0.049862	-0.75647	C	0.791017	0.481537	-0.43962
O	3.520148	0.289207	-1.69575	O	-2.77549	-0.57782	-1.75252
C	4.435831	-1.09179	0.594666	C	-3.87273	0.00555	0.78467
H	-1.23846	-0.55218	-1.67491	H	0.071589	0.771704	-1.21173
C	-2.37941	-0.60249	0.105141	C	0.691878	1.632012	0.59923
C	-3.58529	-0.93832	-0.79801	C	0.60324	2.993593	-0.12255
C	-2.84392	0.180813	1.341133	C	1.805558	1.726266	1.656802
O	-4.59165	-1.69741	-0.13671	O	-0.44305	3.083145	-1.07981
C	0.715458	1.680665	1.888166	C	2.658373	-1.95351	0.941676
H	2.216952	-1.82699	2.005162	H	-1.90749	-0.47584	2.617275

H	0.100336	-2.01718	0.418928	H	-0.15221	-2.23568	1.691136
H	-0.01398	-0.91757	1.766416	H	0.461279	-0.67987	2.172753
H	0.956896	-1.11201	-1.85797	H	-0.88391	-2.58506	-0.76919
H	1.06224	0.625214	-2.14404	H	-0.41692	-1.42612	-2.01369
H	1.320856	1.938379	-0.12726	H	1.402602	-2.87326	-0.52084
H	-0.51955	3.353498	-0.40768	H	1.839477	-1.39447	-2.37633
H	-1.4594	2.506017	0.807794	H	3.404578	-1.42151	-1.5734
H	-2.57499	1.777194	-1.21531	H	2.979596	0.686196	-0.46249
H	-1.07085	1.736749	-2.1144	H	2.244286	0.982637	-2.02798
H	4.573218	-1.60979	1.547405	H	-4.07742	0.226784	1.835563
H	4.921322	-1.67315	-0.19644	H	-4.60345	-0.72973	0.431065
H	4.961888	-0.13255	0.634084	H	-4.04448	0.909721	0.191528
H	-2.00986	-1.57407	0.455748	H	-0.2599	1.505768	1.134617
H	-4.00897	-0.01767	-1.22947	H	1.526415	3.180277	-0.68274
H	-3.24933	-1.56027	-1.63474	H	0.517897	3.793247	0.631106
H	-3.58025	-0.40681	1.899203	H	1.557895	2.493451	2.398643
H	-2.02405	0.398221	2.029496	H	1.957587	0.789045	2.196996
H	-3.31099	1.134663	1.069616	H	2.766568	2.008266	1.213772
H	-5.0913	-1.09816	0.430975	H	-1.28084	2.936676	-0.62037
H	0.85307	2.740125	2.130684	H	3.53267	-2.54766	0.65508
H	1.621311	1.152398	2.197666	H	2.20161	-2.44615	1.805231

H	-0.11632	1.318748	2.502233	H	3.024	-0.97601	1.270197
**1a-k**				**1a-l**			
C	-2.57392	0.11956	0.535111	C	-2.66051	0.255991	0.466682
C	-1.72833	-0.0338	1.571535	C	-1.90396	-0.11635	1.51651
C	-0.4569	-0.84147	1.541737	C	-0.67333	-0.98174	1.437263
C	0.093676	-1.11561	0.125555	C	-0.0318	-1.05724	0.03448
C	-1.12197	-1.57166	-0.72509	C	-1.20111	-1.28866	-0.96004
C	-2.26954	-0.57171	-0.74476	C	-2.29301	-0.23082	-0.88832
C	1.167755	-2.25652	0.087636	C	0.975343	-2.24787	-0.12
C	1.926479	-1.97941	-1.22589	C	1.854826	-1.81056	-1.3077
C	2.082632	-0.45058	-1.25118	C	2.096304	-0.31335	-1.05966
C	0.784354	0.122102	-0.61074	C	0.779177	0.23845	-0.43692
O	-2.94127	-0.38367	-1.75047	O	-2.87427	0.163543	-1.89073
C	-3.82099	0.958618	0.580513	C	-3.86753	1.146975	0.569361
H	0.109707	0.385292	-1.43519	H	0.188577	0.670785	-1.25489
C	0.975882	1.453909	0.170525	C	0.962939	1.409533	0.575798
C	1.104601	2.614403	-0.83853	C	1.176773	2.751821	-0.15336
C	2.13698	1.508099	1.177226	C	2.058856	1.230672	1.63895
O	1.143258	3.898114	-0.22449	O	2.430021	2.868597	-0.82143
C	2.129582	-2.30783	1.285432	C	1.830529	-2.57102	1.115935
H	-1.96694	0.451112	2.518343	H	-2.19149	0.217773	2.513796

H	-0.65881	-1.80753	2.032913	H	-0.95833	-2.00051	1.746915
H	0.287802	-0.35296	2.175917	H	0.04579	-0.64817	2.190624
H	-1.52435	-2.50515	-0.30272	H	-1.68133	-2.25189	-0.72905
H	-0.84931	-1.78211	-1.7623	H	-0.86075	-1.34305	-1.99725
H	0.656044	-3.22658	0.04157	H	0.413091	-3.15676	-0.37005
H	1.336852	-2.31799	-2.08507	H	1.320348	-1.96036	-2.25246
H	2.886727	-2.50392	-1.27342	H	2.785703	-2.38349	-1.37418
H	2.960715	-0.16587	-0.6637	H	2.933772	-0.18348	-0.36929
H	2.247354	-0.06517	-2.26214	H	2.373811	0.207839	-1.98301
H	-3.95174	1.424556	1.560668	H	-4.0464	1.457885	1.601945
H	-4.70647	0.354623	0.355404	H	-4.76177	0.637929	0.194116
H	-3.78452	1.743561	-0.18155	H	-3.74151	2.039125	-0.05234
H	0.050565	1.649762	0.724787	H	0.002402	1.530694	1.092861
H	0.226287	2.626325	-1.49245	H	1.166915	3.56244	0.583702
H	1.986373	2.459172	-1.48129	H	0.341805	2.933674	-0.84786
H	2.082374	2.438381	1.751559	H	1.968213	2.012314	2.401169
H	2.114674	0.687402	1.898012	H	2.002917	0.266774	2.151255
H	3.115108	1.481593	0.682439	H	3.053892	1.328816	1.197637
H	2.024341	4.022257	0.148765	H	2.398511	2.313008	-1.60994
H	2.833277	-3.13729	1.157573	H	2.487496	-3.41893	0.894977
H	1.604918	-2.47055	2.231615	H	1.22143	-2.84975	1.98095

H	2.721332	-1.39305	1.385293	H	2.469865	-1.73485	1.413762
**1a-m**				**1a-n**			
C	-2.58226	0.171834	0.528241	C	-2.65413	0.223338	0.468951
C	-1.74269	0.01186	1.568526	C	-1.89719	-0.16148	1.513879
C	-0.48616	-0.81854	1.547728	C	-0.66062	-1.0173	1.421611
C	0.065189	-1.10975	0.135404	C	-0.01387	-1.06315	0.019494
C	-1.15543	-1.55322	-0.7159	C	-1.17901	-1.28357	-0.98229
C	-2.28527	-0.5339	-0.74602	C	-2.28068	-0.23767	-0.89415
C	1.122993	-2.26456	0.109463	C	1.000752	-2.24642	-0.15307
C	1.869362	-2.0252	-1.21854	C	1.896007	-1.77241	-1.31572
C	2.037106	-0.49785	-1.28865	C	2.137303	-0.28773	-1.01017
C	0.773562	0.109576	-0.60993	C	0.797229	0.241444	-0.42777
O	-2.95016	-0.33831	-1.75489	O	-2.86856	0.166059	-1.88877
C	-3.81774	1.028628	0.563823	C	-3.86922	1.101679	0.584321
H	0.085559	0.403279	-1.41255	H	0.233798	0.664686	-1.26772
C	1.035041	1.431354	0.168796	C	0.933563	1.420664	0.581187
C	1.258949	2.573609	-0.84533	C	1.109412	2.760775	-0.16165
C	2.174418	1.439046	1.199979	C	2.017593	1.272112	1.662805
O	1.3842	3.854215	-0.23702	O	2.286113	2.849194	-0.96031
C	2.096443	-2.30081	1.298335	C	1.838549	-2.59264	1.089072
H	-1.97709	0.505456	2.512152	H	-2.19007	0.152242	2.516336

H	-0.70806	-1.77865	2.042177	H	-0.94079	-2.043	1.712206
H	0.264861	-0.34189	2.182474	H	0.053695	-0.69437	2.184568
H	-1.57382	-2.47699	-0.28772	H	-1.65308	-2.25466	-0.77144
H	-0.8843	-1.77514	-1.75097	H	-0.83419	-1.31587	-2.01886
H	0.598315	-3.22877	0.089691	H	0.447664	-3.1527	-0.43179
H	1.269748	-2.38403	-2.06224	H	1.371242	-1.88701	-2.27116
H	2.825735	-2.55696	-1.26169	H	2.824391	-2.34878	-1.39154
H	2.94203	-0.20465	-0.74846	H	2.940229	-0.20147	-0.27168
H	2.161625	-0.14119	-2.31578	H	2.453999	0.293195	-1.87884
H	-3.94017	1.511466	1.537012	H	-4.0524	1.394571	1.621484
H	-4.71205	0.432993	0.351612	H	-4.75826	0.590483	0.199739
H	-3.7753	1.799621	-0.21221	H	-3.75083	2.004474	-0.02339
H	0.103119	1.676827	0.699371	H	-0.02968	1.520897	1.097354
H	0.44727	2.571112	-1.58973	H	1.071059	3.585654	0.568468
H	2.197892	2.417127	-1.38821	H	0.276652	2.905396	-0.85751
H	2.252895	2.437762	1.636642	H	1.933251	2.082006	2.396247
H	2.02212	0.723516	2.011521	H	1.941702	0.328258	2.208754
H	3.142975	1.218586	0.739012	H	3.029125	1.313769	1.244072
H	0.536911	4.060511	0.179881	H	3.04675	2.878377	-0.36642
H	2.792095	-3.13826	1.178996	H	2.521196	-3.41615	0.853917
H	1.579226	-2.44271	2.252101	H	1.219019	-2.9159	1.931012

H	2.694996	-1.38865	1.376575	H	2.450923	-1.75207	1.429839
